# ZK-AdapterMark: Privacy-Preserving Provenance Evidence for LoRA Adapters

**DOI:** 10.3390/e28101128

**Published:** 2026-10-14

**Authors:** Yicheng Yu, Wangyu Wu, Renwen Ma

**Affiliations:** 1 School of Artificial Intelligence, Shenzhen Polytechnic University, Shenzhen 518055, China; 2 School of Computer Science, University of Liverpool, Liverpool L69 3DR, UK

**Keywords:** LoRA adapters, model provenance, zero-knowledge proofs, model fingerprinting, binary entropy

## Abstract

Low-rank adaptation (LoRA) fine-tunes a large model with a low-rank update over frozen weights. The update travels as a separate adapter file that is easy to copy, alter, and re-upload; so, a hub may have to decide whether a disputed file derives from a registered adapter. Since an altered copy no longer matches the original byte for byte, the usual tool is a fingerprint, secret measurements of the weights that survive moderate edits. Verifying a fingerprint currently requires disclosing either the measurements, which a copier can then evade, or the possibly confidential weights. ZK-AdapterMark is, to our knowledge, the first to verify a LoRA fingerprint in zero knowledge: a hub confirms that a disputed file passes a registered fingerprint without learning the secret or the weights. The proof is practical because our fingerprint is expressed over the two low-rank factors, making the complete proof circuit up to 44 times smaller than rebuilding the update. The acceptance threshold fixes a relative-entropy bound on the probability of accepting an unrelated adapter, which gives it an information-theoretic footing. Across ten model families, the fingerprint survives moderate quantization, noise, and rank reduction, and no pair among 904 independently trained adapters is accepted.

## 1. Introduction

Low-rank adaptation (LoRA) fine-tunes a large model by training a compact low-rank update over its frozen weights and keeping that update separate from the base model [1]. The update is stored as an adapter file of a few megabytes, and such files are exchanged mainly through model hubs, online repositories where anyone can upload an adapter and anyone can download it. A single hub already lists more than thirty thousand adapters for one image model alone [2]. The hub is therefore the place where an adapter is published, where a copy of it reappears, and where a complaint about that copy is filed. The hub, whether or not it seeks the role, is thus the party that ends up judging such complaints.

Judging such a complaint is not straightforward. Possession of an adapter file gives the recipient all of its learned weights. A copy can therefore be renamed, quantized, refactorized, reduced in rank, or merged with another update, and then uploaded again under a new name. None of this happens where the hub can see it. When a dispute arises, the hub holds only two items: the newly uploaded file and, at most, a record that the original owner registered before releasing the adapter. From these, it must decide whether the uploaded file derives from the registered one.

The simplest tool, a hash of the file, answers that question only when the bytes are unchanged, and even mild numerical changes break it while leaving most of the learned update intact. Model ownership research therefore relies on fingerprints instead, secret measurements of the weights whose outcomes stay stable under such changes; when the measurements are derived from a secret key, the fingerprint is keyed. Existing schemes, however, check the fingerprint in the clear. Either the hub receives the secret measurements, after which a subsequent copier can adjust the adapter to evade them, or it receives the adapter weights, which a licence may require the parties to keep confidential. The hub needs evidence about one particular file, and obtaining that evidence should neither use up the secret nor expose the weights.

A convincing answer has three parts, and we first name the parties involved. The claimant is the party who asserts that the adapter is theirs. The registry is a service that keeps a signed, time-ordered log of what claimants have registered. The custodian is a neutral party that receives the disputed file and vouches for exactly which bytes were examined. The verifier is the hub, or anyone else who wants to check the outcome. First, the fingerprint must have been fixed in the registry before the disputed file appeared; otherwise, a claimant could design measurements after the fact that happen to match a file they want to claim. Second, the match must be computed on the file the custodian actually received; otherwise, a proof could be produced from some other adapter and presented as if it concerned the disputed one. Third, the computation must reveal neither the secret measurements nor the weights, for the reasons above. A similarity score alone satisfies none of these, because it says that two objects are similar but not when the test was fixed, which file was tested, or what was disclosed in computing it.

Earlier studies provide several of the needed components. Model watermarking and fingerprinting offer signals that can remain recognizable after certain changes to a model [3,4,5,6]. Chain & Hash considers the time order of competing registrations [7]. HuRef protects a private model comparison, while PVMark conceals the secret detector used for public watermark verification [8,9]. Research on proofs for LoRA also demonstrates that the factorized structure of an adapter can reduce verification cost [10,11,12]. Each of these addresses one of the three parts. None of them binds a test registered in advance to a file authenticated by a custodian while keeping the match computation private.

**Figure 1:**
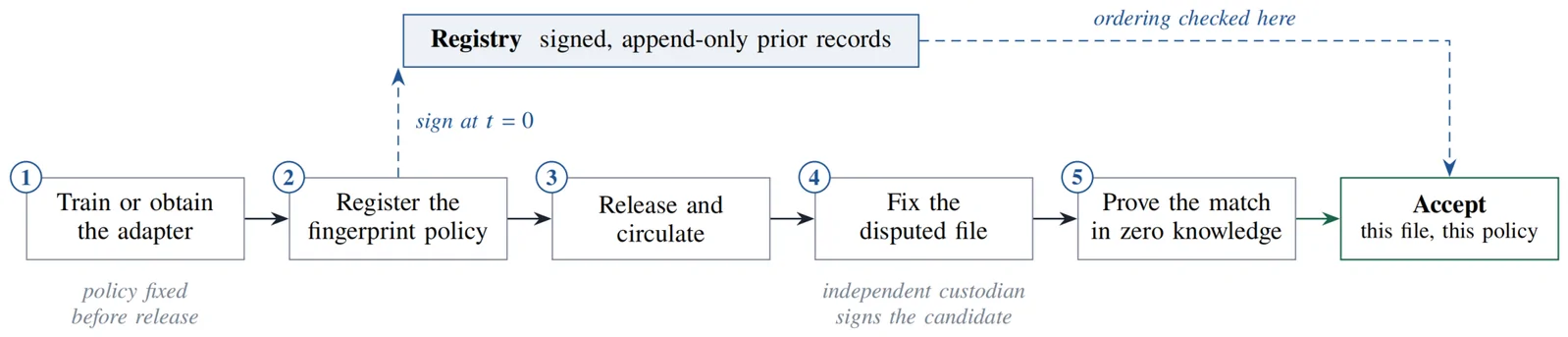
Evidence collected before and after release. The registry first signs an ordered record of the claimant’s fingerprint policy. If a later file is questioned, a custodian authenticates that file independently. Layer proofs are then checked against both records and the policy recorded at registration. The public verifier receives the decision, but not the fingerprint key or adapter factors.

ZK-AdapterMark provides all three parts. It enables the hub to confirm that a disputed file passes a fingerprint registered before release while learning nothing beyond that verdict and the outcome of each layer. The sequence is illustrated in Figure 1. Before release, the claimant deposits with the registry a commitment to the fingerprint, a cryptographic digest that fixes the secret measurements without revealing them, together with the acceptance policy, and the registry signs and time-stamps the record. When a file is disputed, the custodian computes the digest of the received bytes, decodes the two factor matrices by a fixed procedure, and commits to them. A zero-knowledge proof then shows that the committed factors pass the committed fingerprint. The verifier checks the two signed records and the proof together; so, an accepted outcome refers to the specific file the custodian examined and to the policy signed before release. The verifier never sees the fingerprint key or any confidential factors. The prover receives them to construct the proofs and must not disclose them, as Section 2.4 explains.

A zero-knowledge proof requires the fingerprint check to be written as an arithmetic circuit, and the size of that circuit determines how long proving takes. A straightforward circuit rebuilds the dense update even though a LoRA file stores only two narrow factor matrices, and for realistic layer widths that intermediate has millions of entries. We instead apply each measurement to the two factors separately and combine the two short results. This is only a reassociation of the same scalar, but it changes what the circuit has to construct. The computation runs over the two factors and never allocates the full rectangular update, which shrinks the circuit by more than an order of magnitude, and increasingly so for wider layers. Proving remains a server task measured in seconds, while the hub verifies in milliseconds.

A fingerprint is useful only if it survives the edits a copier is likely to make and still rejects adapters unrelated to the registered one. We tested both sides on real adapters. Across 148 adapters from ten model families, quantization to three bits, moderate noise, and halving of the rank leave almost every adapter accepted. Among 904 adapters trained independently from one base model, no pair is accepted, and none of the more than half a million random controls reaches the threshold of a single layer. Among the edits made without regard to the fingerprint, the merge that gives the registered and the foreign update equal weight marks the boundary. We report where this boundary lies rather than averaging over it, because it is the setting a deployment has to choose deliberately. We also give the acceptance threshold an information-theoretic interpretation. It bounds the probability of accepting an unrelated adapter through relative entropy. For a candidate that agrees with the registered bits more often than chance, it is also the minimum amount of information each fingerprint bit must carry about the registered adapter.

The work reported here has three main components:

- A protocol that combines a prior registration, a custodian-authenticated candidate, per-layer proofs, and an adapter policy into one public verification decision.
- A proof relation that evaluates the fingerprint measurements from the stored LoRA factors, which avoids dense reconstruction and keeps the key and confidential factors out of the public transcript.
- Measurements of circuit cost across layer width, rank, and fingerprint length, of proving and verification time, and of fingerprint behavior along transformation sweeps, together with an information-theoretic interpretation of the threshold.

The rest of this paper proceeds as follows: Section 2 specifies the records and the verification procedure. Section 3 and Section 4 define the private match and show how it is evaluated from LoRA factors. Section 5 analyzes security and derives the acceptance threshold. Section 6 reports the measurements. Section 7 reviews related work, and Section 8 concludes and states the limitations. Appendix D and Appendix E collect the protocol syntax, the trust analysis, the proofs, and additional results.

## 2. Evidence Protocol for a Disputed Adapter

A dispute in ZK-AdapterMark is decided from evidence produced at two different times, and the order of the two matters. The first event is registration. It takes place while the claimant still possesses the original adapter and before the file is distributed. The second event is the fixing of the candidate. The disputed file may appear weeks or months later, when it is obtained by a model hub or an auditor, and at that point, the custodian records the file independently. This step fixes the candidate before proving begins and prevents the claimant from replacing it with another adapter that is easier to match. Everything the verifier later checks traces back to one of these two events and must satisfy the three requirements stated earlier: (a) the test was fixed before the disputed file appeared, (b) the match was computed on the file the custodian actually received, and (c) neither the secret measurements nor the weights were disclosed.

ZK-AdapterMark links the two events through three pieces of evidence. (a) The *prior record* is what the registry signs at registration. It holds a commitment to the fingerprint of each selected layer, the threshold of each layer, and the rule that turns the layer outcomes into a decision about the whole adapter. Each layer is labelled with a *context* that names the model and the layer it belongs to; so, a commitment cannot be read under another model. Section 3 lists its fields. (b) The *candidate record* is what the custodian signs during a dispute. It contains a digest of the file received and commitments to the factor matrices recovered from that file. (c) The *private match proof* is produced for these committed factors under the previously registered test. The same contexts appear in all three pieces; so, the verifier can tell that the proof concerns the registered policy and the file in the custody record.

The three pieces are not interchangeable, and each answers one of the three requirements. The registry’s signature shows when the test was fixed, which is requirement (a). The custodian’s record shows which file was tested, requirement (b), provided the custodian followed the declared decoding. The proof shows that this file passes the registered test without revealing the test or the weights, requirement (c). The first two rest on signed records, which the verifier checks directly. The proof covers only the arithmetic on the committed values.

### 2.1. Threat Model

Before stating what an adversary may do, we recall what each role signs, since the signatures are what an adversary must defeat. The registry signs the claimant’s policy record and fixes its position in the log. In a dispute, the custodian decodes the file and signs its digest together with commitments to the decoded factors. A fifth role, the *prover*, receives the secret inputs, namely the fingerprint key and the factors, and constructs one proof per layer. The verifier checks the records and the proofs and computes the decision for the adapter. One organization may perform several of these functions, but the signature labels still indicate which role is responsible for each record.

The adversary considered here is not limited to passive observation. It may inspect the protocol and the public registry, redistribute an adapter, alter its factors, make a competing claim, or submit selected files for testing. The security of the cryptographic part relies on the assumed properties of the signature, commitment, and zero knowledge schemes. Some responsibilities remain operational: the registry must preserve the order of accepted records, and the custodian must apply the canonicalization procedure of Section 4.4 to the file in its possession. Both operations are signed and logged; so, an auditor can examine whether they were performed correctly, but neither is treated as a fact proved by the circuit. Section 2.4 examines what remains of each guarantee when the registry, the custodian, or other parties deviate from the protocol.

The evidence addresses non-evasive reuse. The reuser deploys the adapter directly, quantizes, prunes, or merges it for deployment, or continues training it on task data, but does not aim to remove the fingerprint. Proposition 3 relates such edits to the angle between the edited and the registered update. When its conditions hold, an update that stays well within 45° of the registered update keeps its expected agreement above the threshold. An adversary that sets out to remove the fingerprint is outside the threat model of this paper. Section 6.6 measures three such strategies. The first perturbs the factors along input directions of low variance. The second continues training with the goal of lowering the similarity to the registered update. The third distils the outputs of the stolen model into a freshly initialized adapter. All three remove the fingerprint at a small cost in utility, as fingerprints and watermarks read from the weights generally allow [13]. Adversaries on the side of the claimant remain in scope, and Section 6.6 shows that a false claim fails. Appendix B.2 discusses this boundary in relation to existing methods.

### 2.2. Protocol Workflow

With the roles fixed, the workflow runs in three stages: registration, fixing the candidate, and proving. Registration starts with a screening step that the claimant performs on its own adapter. A layer whose update is nearly zero gives no reliable signs and is left out, and Section 6.3 found such layers in a public adapter. The registry cannot see this step, and the proof does not check it. Skipping it weakens only the claimant’s own evidence, since an unrelated candidate matches a degenerate layer on about half of its probes. For every layer that remains, the claimant derives the secret measurement directions from a key and creates two commitments. One binds the original factors. The other binds the fingerprint test, that is, the key and the reference responses, the bits the measurements produce on the original adapter. The reference responses are not accepted merely as values supplied by the claimant. A consistency proof demonstrates that they were calculated from the factors in the factor commitment. Once this proof and the registration requirements have been checked, the registry records the layer thresholds and the rule for combining their outcomes. The factors and fingerprint keys are not disclosed to the registry.

The candidate side of the protocol begins only after the custodian receives a particular file. The custodian computes a digest of its bytes and applies the canonical decoder specified by the relevant circuit version. It then commits to the extracted factor matrices and signs these commitments together with the digest and layer contexts. Any later match proof must refer to the same commitments. Testing another adapter would therefore require the custodian to issue a different candidate record.

In the proving stage, the prover evaluates the registered measurements against each committed layer and returns one proof per layer. Public inputs are reconstructed from the signed records, not accepted as free-form values supplied with the proof. After checking the individual proofs, the verifier counts their outcomes using the rule in the prior record. This is where the numerical result becomes a decision about the recorded candidate rather than about an unspecified set of matrices.

Appendix D.1 states this workflow as five algorithms and writes out the acceptance condition, Equation (A2). In short, the verifier accepts when both records carry valid signatures, they name the same contexts, the signed policy is admissible, and enough layers carry a valid proof. A missing or invalid proof leaves its layer unestablished, which is weaker than evidence that the layer fails.

### 2.3. Public and Confidential Candidate Modes

One detail of the workflow depends on whether the disputed adapter is itself public. When the adapter can be downloaded, anyone can derive its factors and check the candidate commitment; secrecy is needed only for the key and registered responses. A licensed adapter may instead remain in the custodian’s environment. In that case, the custodian authorizes the prover to use the extracted factors, while the public verifier receives only their signed commitments and the layer proofs.

Neither deployment mode places the fingerprint key in the public transcript. When the adapter is confidential, its factor matrices are also withheld from the verifier. The prover is an exception: it needs access to those matrices in order to construct the witness. Confidential mode therefore restricts the weights to an authorized proving environment instead of claiming that they remain secret from the party generating the proof.

### 2.4. Trust Assumptions and Compromised Parties

A verdict depends on several parties, any of which can deviate from the protocol. Three guarantees follow from cryptographic assumptions. An accepted proof implies that the committed candidate passes the committed test, provided the setup of its circuit was honest. The verifier learns nothing about the key or the reference responses beyond the public records and the layer outcomes. In the confidential mode it also learns nothing about the candidate factors. Both require hiding commitments and a prover that follows the protocol. Two guarantees rest on parties. The registry fixes when the test was registered, and the custodian fixes which file was tested. A dishonest custodian can commit to factors of another file. Any verifier detects this in the public mode by recomputing the commitment from the downloaded file, and in the confidential mode, independent custodians or attested decoding reduce the dependence. A dishonest registry can backdate a record, which a witnessed transparency log makes detectable. Appendix D.2 names the five guarantees G1 to G5, examines each party, including the prover and the setup, and lists what each coalition can break. If the claimant, the registry, and the custodian all collude, they can produce an accepting bundle for any file. A deployment should therefore select registries and custodians whose interests are independent of the claimant’s.

## 3. Private Fingerprint Matching

The protocol of the previous section says when the fingerprint test is fixed and which file it is applied to. This section defines the test itself. Each secret measurement, which we call a *probe*, produces a single scalar from the adapter, and registration retains only the direction of that scalar, that is, whether it lies above or below zero. Small numerical changes to an adapter will often leave this direction unchanged even though the stored factor values are no longer identical. An unrelated update, by contrast, has no reason to reproduce any particular direction. Under the *fair-bit assumption*, which treats each of its directions as a fair coin flip relative to the registered one, it is expected to agree with about one half of the registered signs. The reference bit string is fixed in the prior record. During a dispute, the circuit calculates the corresponding bits from the candidate factors and counts the positions at which the two strings agree.

### 3.1. Directional Responses

Let a LoRA layer store $B\in R^{d_{\mathrm{out}}\times r}$ and $A\in R^{r\times d_{\mathrm{in}}}$. Each probe is a keyed random projection of the update with ±1 entries. The *i*th pair of sign directions is obtained from a keyed generator:

(1) $$\left(u_{i},v_{i}\right)=G_{k}\left(\mathrm{ctx},i\right)\in {\left\{-1,+1\right\}}^{d_{\mathrm{out}}}\times {\left\{-1,+1\right\}}^{d_{\mathrm{in}}}.$$

The directional score and its one-bit response are:

(2) $$s_{i}=u_{i}^{T}BAv_{i},\;b_{i}=1\left[s_{i}\geq 0\right].$$

For *m* measurements, the bits $b_{1},\ldots ,b_{m}$ form the layer fingerprint. Both direction vectors are derived from the secret key together with the layer context. Reusing a response string under another model or layer therefore does not recreate the same test.

A score arbitrarily close to zero is a special case since fixed-point rounding alone may reverse its sign. The registration procedure consequently tests the score margins for every proposed layer and key. A probe set that does not meet the recorded margin condition is discarded and a fresh set is derived from a new key in the same way. Appendix D.4 gives a margin that suffices for the integer encoding of Section 4.4. The claimant performs this test before registration. It conditions the probes on the registered adapter and leaves a probe of an unrelated candidate about as likely to agree as to disagree. After the registry accepts a set, neither its coordinates nor their order may be changed for a later candidate.

### 3.2. Registration and Candidate Matching

Registration then creates separate commitments for the adapter and its fingerprint test. The *factor commitment* binds the claimant to the original factors *B* and *A*. The *test commitment* binds the secret key and reference bits together with the fingerprint length, threshold, and context. The context introduced in Section 2 is given here in full: it identifies the base model, adapter, complete parameter path, matrix dimensions, rank, signed integer representation, and circuit version. These fields prevent the same commitment from being interpreted under a different model or circuit configuration.

The registry does not receive the openings of either commitment. Instead, it verifies a consistency proof in which the reference responses are calculated again from the committed factors and the hidden test data. This calculation is necessary because a reference string supplied without such a link may have no relation to the registered adapter. A dishonest claimant could otherwise choose the string first and later look for a candidate that happens to reproduce it. The policy record is signed only after the consistency proof and the claimant authentication have been accepted. Layer eligibility rests on the claimant’s screening, which the registry cannot see.

For a disputed layer, applying the committed test to the candidate gives $b_{1}^{\prime },\ldots ,b_{m}^{\prime }$. We count matching positions as follows:

(3) $$c=\sum_{i=1}^{m}1\left[b_{i}=b_{i}^{\prime }\right].$$

The layer passes when c≥τ, where the prior record supplies τ. We use m=128 and τ=96 in the experiments. Each of the six selected layers has its own context and key. The outer verifier, rather than any single layer circuit, requires five passing layers for an adapter-level acceptance.

Registration and dispute check the same computation and differ only in how many bits must agree; so, one relation $R_{\mu }$ serves both through a mode bit μ. With μ=1, used at registration, all *m* responses must agree. With μ=0, used for a candidate, the recorded threshold suffices. The relation also requires both commitments to open to the values used in the computation, and Appendix D.3 states it in full. It says nothing about custody or registry order, which the verifier checks from the signed records.

### 3.3. Threshold as a Policy Operating Point

The threshold τ is the one parameter the relation leaves open, and its value belongs to the fingerprint policy rather than to the security configuration of the proof system. Setting τ=m accepts only a complete match between the two response strings. Such a rule may reject a related adapter when rounding or an equivalent refactorization changes a small number of signs. Reducing τ allows more modified versions to pass, although it also increases the range in which an unrelated candidate may be accepted. For this reason, the selected value is included in the signed registration record and cannot be adjusted after the disputed file is known. Section 5 estimates the probability that an unrelated adapter reaches a given threshold, which is distinct from the cryptographic soundness of the proof system, and Section 6 evaluates the threshold used in our experiments.

## 4. Factor-Aware Proof Construction

**Figure 2:**
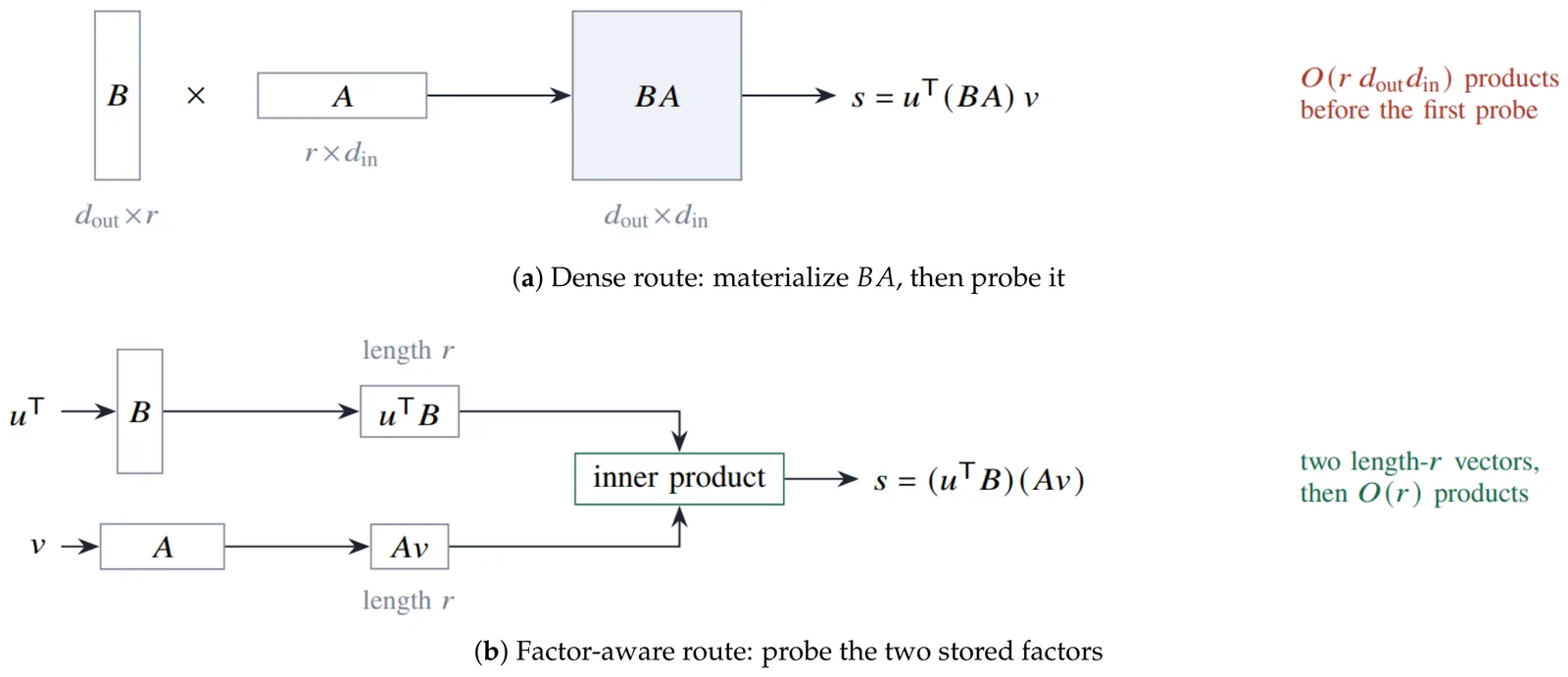
Two evaluations of one directional score. Route (**a**) forms all $d_{\mathrm{out}}d_{\mathrm{in}}$ entries of BA before applying the probe. Route (**b**), used in our circuit, first projects *B* and *A* and then combines the two length-*r* vectors. Associativity gives $u^{T}\left(BA\right)v=\left(u^{T}B\right)\left(Av\right)$; so, the numerical response is unchanged even though no dense update is built.

The relation of the previous section is proved in zero knowledge by expressing it as an arithmetic circuit, a fixed network of additions and multiplications over a finite field. The number of constraints in that circuit sets the proving cost. A straightforward circuit would reconstruct the dense update before it evaluates even one fingerprint probe. We instead keep the LoRA factors separate throughout the calculation. Figure 2 illustrates where the two evaluation routes diverge, although they return the same scalar.

### 4.1. Avoiding Dense Reconstruction

For one LoRA layer, the represented update is:

(4) ΔW=BA,

where $B\in R^{d_{\mathrm{out}}\times r}$, $A\in R^{r\times d_{\mathrm{in}}}$, and typically $r\ll d_{\mathrm{in}},d_{\mathrm{out}}$. Materializing this matrix in a circuit costs about $rd_{\mathrm{out}}d_{\mathrm{in}}$ multiplications, and that cost is paid before Equation (2) supplies a response.

Associativity allows the probe to be applied before the product is formed:

(5) $$u^{T}BAv=\left(u^{T}B\right)\left(Av\right).$$

The two quantities in parentheses have length *r*. Their inner product is exactly the dense directional score, but the amount of data traversed is proportional to $r(d_{\mathrm{out}}+d_{\mathrm{in}})$ rather than $d_{\mathrm{out}}d_{\mathrm{in}}$.

**Proposition** **1** (factor-aware equivalence and cost)**.** *Suppose the signed fixed-point bounds rule out modular wrap-around. Equation (5), evaluated in the circuit field, then gives the same scalar as explicit construction of BA. With signs known in advance, a factor-based probe uses $O(r\left(d_{\mathrm{out}}+d_{\mathrm{in}}\right))$ additions and O(r) general multiplications. With secret signs, as in our circuit, it uses $O(r\left(d_{\mathrm{out}}+d_{\mathrm{in}}\right))$ multiplications. The dense-first alternative uses $O(rd_{\mathrm{out}}d_{\mathrm{in}})$ multiplications merely to form the update.*

Appendix D.5 gives the proof.

Because the signs are secret, each term of the two projections costs one product of a sign bit and a factor entry. The kernel of the circuit therefore has $m\left(r(d_{\mathrm{out}}+d_{\mathrm{in}})+140\right)$ constraints, as Appendix A reports. This count is still linear in the layer dimensions, whereas the dense route grows with their product.

### 4.2. Circuit Pipeline

The probe evaluation is the core of the circuit, but not all of it. In the circuit, the supplied B,A pair is first checked against the candidate commitment, that is, the circuit recomputes the commitment from the supplied factors and requires it to equal the public value. A second such opening binds the key and reference responses to the test commitment and its context. The probe signs are then derived from the key with Poseidon, a hash function designed for a low constraint count inside arithmetic circuits [14], with distinct input tags for each use. Equation (5) produces the candidate responses. What remains is to compare signs, accumulate the matches, and enforce the final line of Equation (A4).

The input tags, known as domain separation, matter more than their size suggests. The context prevents a layer proof from being read under another model or circuit version, and distinct role tags keep the *u* and *v* streams apart. Each hash output supplies the signs of 252 consecutive coordinates of a direction, and Appendix D.4 explains why its two highest bits are left out. The mode bit selects exact or threshold matching. Boolean and range checks then connect the field computation to the binary responses and signed fixed-point interpretation required by the protocol.

### 4.3. Implemented Per-Layer Circuit

The implemented circuit enforces the complete relation of Equation (A4) for one layer. Its public inputs are the mode bit μ, the context digest ctx, the factor commitment $C_{F}$, the test commitment $C_{\mathrm{wm}}$, and the threshold τ. Its private inputs are the two factors, the key, the reference bits, and the two blinding values. Dimensions and the circuit version are compiled into the circuit; so, each supported shape is a separate circuit. Table 1 lists the checks the circuit performs and the constraints each one costs.

The context is bound in two places. The circuit first derives a layer key $k_{\mathrm{ctx}}=\mathrm{Poseidon}\left(k,\mathrm{ctx}\right)$ and generates every probe seed from $k_{\mathrm{ctx}}$; so, the probe directions of one layer are not reproduced under another context. It then hashes ctx into both commitments, together with a tag that distinguishes the factor commitment from the test commitment and, in the factor commitment, the mode bit. A commitment made at registration therefore cannot be presented as a candidate commitment. The blinding value of each commitment is its last input. In the confidential mode the custodian draws $\rho_{F}$ at random and keeps it private, which makes the candidate commitment hiding. In the public mode $\rho_{F}$ is published with the candidate record; so, anyone can recompute the commitment from the downloaded file. The claimant draws $\rho_{\mathrm{wm}}$ at registration and keeps it with the key. At registration the claimant also draws $\rho_{F}$ for the commitment to its own factors and keeps it private. Each blinding value is a random 248-bit number. If Poseidon is modeled as a random function, a commitment whose blinding value stays secret is hiding. The test commitment and the registered factor commitment are therefore always hiding. In the public mode the candidate commitment is deliberately not hiding, since the file itself is public.

The circuit also checks that its private inputs have the meaning the relation assumes. The mode bit and every reference bit satisfy b(b−1)=0. This constraint is needed for soundness. Without it the reference bits enter the circuit only through their packed value $\sum_{i}b_{i}2^{i}$ in the test commitment, and this single linear equation has many solutions in the field that are not bits. A prover could choose such values to raise the agreement count above its true value. The reference bits are packed in blocks of 128 per field element; so, the packing is one-to-one for every *m*, and the test commitment also contains *m*. Every factor entry is range-checked to a signed 24-bit integer in $\left[-2^{23},2^{23}\right)$, and Lemma A1 in Appendix D.5 shows that no further range check is needed. Outside the circuit the verifier checks the signatures, the context equality, the admissibility of the policy, and the adapter-level rule, all on public values. One proof per layer lets the verifier see which layers lack a valid proof.

The lemma bounds every partial sum by $2^{81}$ in absolute value for every shape counted in Section 6.8, far below half of the field order; so, the field computation equals the integer computation. The circuit extracts the sign of a score *s* over N=128 bits, as the top bit of the 128-bit decomposition of $s+2^{127}$, which satisfies the lemma with a wide margin.

### 4.4. Signed Encoding and Proof Backend

Two practical matters remain: how the factor values are encoded, and which proof system is used. The factor matrices read from a LoRA file are represented with floating point values and cannot be used directly by the arithmetic circuit. Before they enter the witness, the factors are brought into a balanced form, computed from QR and singular value decompositions, that depends only on their product BA. Two files that store the same update as (B,A) and as $\left(BQ,Q^{-1}A\right)$ therefore yield the same encoded factors, up to ties among singular values and floating-point precision. Each balanced factor tensor is then scaled by a power of two and rounded to integers whose largest magnitude lies between $2^{21}$ and $2^{22}$, inside the signed 24-bit range. A positive scale changes no response sign, and the rule is deterministic; so, anyone who decodes the same file obtains the same integers. Appendix D.4 gives the construction, the rounding rule, the encoding of negative integers, and a margin that keeps the signs stable. The serialization rule and numerical bounds are versioned as well. Consequently, the factors used at registration and those extracted during a dispute are interpreted by the same conversion procedure. The timed prototype of Section 6 encoded the stored factors directly. The balanced form is computed outside the circuit, before the commitments; so, it leaves the circuit unchanged.

This conversion introduces two issues that must be checked separately. First, factor values and accumulated sums must remain within the permitted range; otherwise, modular reduction in the field could produce an apparently valid result that does not correspond to the intended integer calculation. The range checks and Lemma A1 settle this inside the circuit. Second, a response close to zero may change sign after rounding. As described in Section 3, the registration procedure examines the response margins and rejects such probes before they become part of the fingerprint policy.

The prototype relation was written in Circom [15], a language for describing arithmetic circuits, and proved with Groth16 over the BN254 curve [16]. Groth16 produces proofs of a few hundred bytes that are verified with a fixed number of pairing operations, which is why we chose it: the hub’s verification cost does not grow with the circuit. Each supported circuit shape has its own setup material, which Section 6.8 discusses. Each selected layer also receives its own proof; so, the verifier can see which layer failed and can combine the layer outcomes with the decision rule stored in the registration record.

## 5. Security Analysis

We analyze three properties of an accepted verdict. It is bound to one authenticated candidate and one policy that predates it, which we call evidence binding. It reveals nothing beyond the verdict itself and the outcome of each layer, which is zero knowledge. And it is unlikely to be reached by an adapter unrelated to the registered one, which we call selectivity. The first two are cryptographic and follow from standard assumptions on the signature, commitment, and argument systems; Proposition 2 states them and Section 5.2 discusses the assumptions. Selectivity is statistical rather than cryptographic. Section 5.3 relates the agreement of one probe to the angle between two updates, Section 5.4 derives selectivity from it, and that derivation is the source of the acceptance threshold used in the experiments. Section 5.5 then considers an adversary who modifies an adapter with the fingerprint in mind.

### 5.1. Conditional Evidence Binding

**Proposition** **2** (Conditional evidence binding)**.** *Consider a bundle accepted under Equation (A2). Assume unforgeable registry and custodian signatures, binding commitments under the documented encoding, and knowledge soundness and zero knowledge of the proof system. Then, except with negligible cryptographic failure probability:*

*(i)*  *A valid registration proof binds $C_{\mathrm{wm}}$ to a key and responses derived from factors opening the original-factor commitment $C_{F}$ with μ=1;*
*(ii)*  *The custodian authenticates $D^{*}$ and $C_{\mathrm{cand}}$ under the same context;*
*(iii)*  *There exist candidate factors opening $C_{\mathrm{cand}}$, together with a key and responses opening $C_{\mathrm{wm}}$, for which $R_{0}=1$, that is, the dispute-mode relation of Equation (A4) holds.*

*In the zero-knowledge model, verification discloses no witness component beyond information implied by the public statement and its outcome.*

Appendix D.5 sketches the proof.

### 5.2. Soundness, Privacy, and Binding

Proposition 2 relies on four cryptographic assumptions and one operational requirement. We state what each provides.

*Soundness:* Completeness is immediate: if the registered factors generated the reference string and the candidate crosses the recorded threshold, an honest prover holds a witness for $R_{\mu }$. Soundness follows from the knowledge soundness of the argument system together with the binding of the commitments. For any accepted proof, the extractor yields openings of the factor and test commitments and a witness satisfying the keyed probe computation and the threshold, and binding rules out substituting different openings after the records were signed. The extracted witness is a vector of field elements. Its interpretation as adapter tensors is fixed by the versioned serialization rule, the signed-integer encoding, and the range constraints. Without the range constraints, an identity in the field could correspond to an integer computation that wrapped around. The circuit enforces these range constraints and the Boolean constraints on the mode and reference bits. The extracted factors are therefore bounded integers, the extracted responses are bits, and Lemma A1 identifies the field computation with the integer one.

*Zero knowledge:* The zero-knowledge property of the argument system guarantees that the verifier obtains no information other than the public statement and the verdict of each layer. The key, the reference bits, and the factors therefore remain hidden from the verifier, up to what the layer outcomes themselves imply. For the full protocol this also requires hiding commitments, fresh blinding values, and a prover that follows the protocol. In confidential mode, the factors are additionally withheld from the public interface and are available only to the authorized prover.

*Binding of records:* Existential unforgeability of the registry and custodian signatures binds the prior record and the candidate record to their issuers. The context embedded in every commitment binds the commitment to one model, layer, dimensions, encoding, and circuit version so that a proof cannot be reinterpreted under another configuration. Canonical decoding binds the candidate commitments to the file digest: the same bytes decode to the same factors and, with the same blinding value, to the same commitments.

*Policy enforcement:* The adapter-level rule is evaluated by the verifier outside the circuit but within the signed acceptance procedure of Equation (A2). Distinct contexts and keys per layer prevent a proof for one layer from being replayed as another layer’s result, and the verifier rejects duplicate layer positions before counting passes.

*Trusted setup:* Groth16 requires a structured reference string, and its soundness holds only if that setup was generated correctly. The circuit digest, setup transcript, proving-key identifier, and verification key must therefore be published together under one version. A transparent argument system removes this requirement at the cost of larger proofs and slower verification.

### 5.3. Agreement and the Angle Between Updates

The fingerprint must pass modified copies of the registered adapter and reject unrelated adapters. Both requirements depend on how likely one probe is to agree between two different updates. Let X=BA be the registered update of a layer and $Y=B^{\prime }A^{\prime }$ the update of a candidate for the same layer. A probe (u,v) agrees when $u^{T}Xv$ and $u^{T}Yv$ have the same sign. The probe directions come from a keyed pseudorandom generator, and we model their entries as independent uniform signs. Write ${\langle X,Y\rangle }_{F}=\mathrm{tr}\left(X^{T}Y\right)$ for the Frobenius inner product and

(6) $$\theta =\arccos\frac{{\langle X,Y\rangle }_{F}}{{∥X∥}_{F}\;{∥Y∥}_{F}}$$

for the angle between the two updates. The angle can be computed from the factors without forming either product, since ${\langle BA,B^{\prime }A^{\prime }\rangle }_{F}=\mathrm{tr}\left(\left(B^{T}B^{\prime }\right)\left(A^{\prime }A^{T}\right)\right)$ involves only r×r matrices.

**Proposition** **3** (Agreement and update angle)**.** *Fix u, and let $x=X^{T}u$ and $y=Y^{T}u$. Suppose that no single coordinate carries a non-negligible share of ${∥x∥}^{2}$ or ${∥y∥}^{2}$. Then, the pair $\left(u^{T}Xv,\;u^{T}Yv\right)=\left(x^{T}v,\;y^{T}v\right)$ is approximately bivariate normal with correlation $\rho_{u}=\langle x,y\rangle /\left(∥x∥\;∥y∥\right)$, and the probe agrees with probability:*

(7) $$p_{u}\approx 1-\frac{\arccos\rho_{u}}{\pi }.$$

*The agreement probability of one probe is the average of $p_{u}$ over u. The expectations satisfy $E_{u}\langle x,y\rangle ={\langle X,Y\rangle }_{F}$ and $E_{u}{∥x∥}^{2}={∥X∥}_{F}^{2}$. If, in addition, 〈x,y〉, ${∥x∥}^{2}$, and ${∥y∥}^{2}$ stay close to their expectations for most u, then $\rho_{u}\approx \cos\theta$. This requires both updates to spread over many directions of comparable weight. The agreement probability of one probe then becomes:*

(8) $$p\approx 1-\frac{\theta }{\pi }.$$

Appendix D.5 sketches the proof. For updates of low rank, $\rho_{u}$ varies with *u*, and Equation (8) is then only an approximation whose error depends on the updates. Section 6.4 measures this error on real adapters.

Equation (8) has the form of the collision rule of random-hyperplane hashing [17]. That rule holds exactly for directions drawn uniformly from the sphere. Our sign directions have the rank-one form $uv^{T}$ and satisfy it only under the conditions of Proposition 3. Under these conditions, it places both requirements on one scale. A transformed copy changes the update by a small angle and keeps *p* close to one. An adapter trained independently has an update nearly orthogonal to the registered one; so, θ is close to 90° and *p* is close to 1/2. The expected count m(1−θ/π) reaches τ at an angle of 45° for τ/m=3/4. A probe at angle θ then also acts as a binary symmetric channel with crossover probability θ/π; so, it carries $1-H_{2}\left(\theta /\pi \right)$ bits about the registered response, where $H_{2}$ is the binary entropy function. Section 5.4 uses this reading to interpret the threshold.

### 5.4. Statistical Separation from Unrelated Adapters

Proposition 2 guarantees that an accepted proof was computed honestly on the committed candidate. It says nothing about whether that candidate is related to the registered adapter, and this is a different question. Cryptographic soundness rules out proving a statement that is false. Fingerprint selectivity concerns a statement that is true of an adapter that is simply unrelated and happens to cross the threshold. The second question is statistical, and we first separate what follows from the construction under a model of its key generator from what has to be measured. We model the keyed generator as a pseudorandom function so that for a fixed registered layer and a fixed candidate layer the probe directions behave as independent uniform sign vectors. Under this model the *m* agreement indicators are independent and identically distributed, each equal to one with the probability *p* of Proposition 3, and the agreement count is binomial, c∼Bin(m,p). Different layers use different keys and contexts; so, for a fixed pair of adapters, the counts of different layers are independent under the same model. What the construction does not control is the value of *p*, which depends on how the two updates are related. An unrelated adapter that shares no structure with the registered one has θ close to 90° and p=1/2. We call this the fair-bit case. In this case, the probability of at least τ agreements is:

(9) pm,τ=2−m∑j=τmmj.

Entropy measures describe the uncertainty of discrete responses, and machine learning uses them widely [18]. They also give the threshold a direct reading. If disagreements are modeled as independent flips with probability η, the candidate response is the output of a binary symmetric channel with crossover η, and each probe carries

(10) $$I\left(b;b^{\prime }\right)=1-H_{2}\left(\eta \right)$$

bits about the registered response, where $H_{2}$ is the binary entropy function. Write ε=1−c/m for the observed fraction of probes on which the candidate disagrees with the registered string. It estimates η, and $1-H_{2}\left(\varepsilon \right)$ is the corresponding plug-in value. An unrelated adapter corresponds to η=1/2: its responses have maximal entropy, $H_{2}\left(1/2\right)=1$ bit, and carry no information about the registered string. The layer rule c≥τ is the condition ε≤1−τ/m. For a candidate that agrees more often than chance, ε≤1/2, it is equivalent to a plug-in information of at least $I^{*}=1-H_{2}\left(1/4\right)\approx 0.19$ bit per probe at m=128 and τ=96. The test is one-sided, since a candidate whose bits are mostly inverted also carries much information but is rejected. Section 6 plots this quantity along the transformation sweeps and checks the model against the unrelated controls.

The false-accept probability has a matching expression. By the Chernoff bound for a binomial tail,

(11) $$p_{m,\tau }\leq 2^{-m\;D(\tau /m\;∥\;1/2)},$$

where *D* is the binary relative entropy. This one-sided exponent equals $m\;I^{*}$, since $D\left(\tau /m\;∥\;1/2\right)=1-H_{2}\left(\tau /m\right)$; so, the evidence against a chance match, measured in bits as $-\log_{2}p_{m,\tau }$, is at least the information the threshold demands from each probe, summed over the *m* probes. With m=128 and τ=96 the bound gives 24.2 bits, and the exact tail in Equation (9) gives $p_{m,\tau }\approx 6.4\times 10^{-9}=2^{-27.2}$, that is, 27.2 bits. This is the origin of the threshold used in Section 6: τ=96 lies 5.7 standard deviations above the mean of an unrelated adapter, while a related adapter that keeps three quarters of its bits still passes.

Adapters trained from the same base model on related tasks may share part of their update. Their angle to the registered update is then below 90°, and *p* exceeds 1/2. The per-layer false-accept probability becomes the general binomial tail:

(12) PFA(p)=∑j=τmmjpj(1−p)m−j≤2−mD(τ/m∥p),p<τ/m,

which grows quickly with *p*. At m=128 and τ=96 it equals $6.4\times 10^{-9}$ for p=0.5, $2.3\times 10^{-6}$ for p=0.55, $2.6\times 10^{-4}$ for p=0.6, and $1.0\times 10^{-2}$ for p=0.65. By Equation (8), these values of *p* correspond to angles of 90°, 81°, 72°, and 63°.

The adapter-level rule accepts when at least *k* of the *L* registered layers pass. For a fixed candidate with agreement probabilities $p_{1},\ldots ,p_{L}$ the layers are independent, and the number of passing layers follows a Poisson binomial distribution with success probabilities $P_{\mathrm{FA}}\left(p_{1}\right),\ldots ,P_{\mathrm{FA}}\left(p_{L}\right)$. The adapter-level false-accept probability of an unrelated candidate is the average of this tail over candidates:

(13) $$P_{\mathrm{FA}}^{\mathrm{adp}}=E_{\mathrm{cand}}\left[\mathrm{Pr}\left(\sum_{\ell =1}^{L}Z_{\ell }\geq k\right)\right],\;Z_{\ell }∼\mathrm{Bernoulli}\left(P_{\mathrm{FA}}\left(p_{\ell }\right)\right)\;\mathrm{independent}.$$

In the fair-bit case, every $p_{\ell }=1/2$, and the five-of-six rule gives about $2^{-133}$. Dependence between layers enters Equation (13) only through the joint distribution of $p_{1},\ldots ,p_{L}$ over candidates. A candidate that resembles the registered adapter in one layer may resemble it in the others, and the average is then dominated by the most similar candidates. In the extreme case in which all layers share one value *p*, the five-of-six rule gives $4.0\times 10^{-28}$ at p=0.55, $7.6\times 10^{-18}$ at p=0.6, and $5.8\times 10^{-10}$ at p=0.65. Section 6.5 measures the distribution of *p* on independently trained adapters and evaluates Equation (13) with it. All of these values are fingerprint selectivity estimates. They are not cryptographic security levels and should not be substituted for the parameters governing Groth16, signatures, commitments, or the registry.

The number of attempts matters as well. For *q* attempts, a union-bound calculation increases the chance-match upper bound by at most a factor of *q*. The attempts include every key that a claimant derives and tests before it registers, which the registry cannot observe. A registry should therefore authenticate and retain requests, issue fresh contexts, and restrict repeated trials.

### 5.5. Adaptive Adversaries

The transformations of Section 6.3 are edits a copier makes without regard to the fingerprint. An adaptive adversary instead knows the complete scheme: the probe construction, the threshold, the layer policy, and the circuit. It holds the adapter file, the base model, and data from the adapter’s task. It does not know the key. Its goal is a modified adapter that keeps the task utility of the original and fails the registered test.

Without the key the adversary cannot aim at individual probes. From its position every probe is a fresh pair of uniform sign vectors. With the balanced form of Section 4.4, the encoded factors depend only on the update; so, the adversary can lower the agreement only by changing the update. When the conditions of Proposition 3 hold, the expected agreement of a layer is approximately a function of the angle between the modified and the registered update. The expected count then falls below τ once this angle exceeds:

(14) $$\theta^{*}=\pi \left(1-\frac{\tau }{m}\right).$$

This angle is 45° for τ/m=3/4, and the rotation is needed in at least L−k+1 of the *L* registered layers. The binomial spread of the count blurs this boundary by a few degrees in either direction. For updates that violate the conditions, the angle only predicts the agreement. The question is therefore whether an update can be rotated this far while the adapter keeps its utility. The answer depends on the model and the data; so, Section 6.6 measures it for seven strategies:

1. Fine-tuning with a penalty on the cosine similarity between the new and the registered update;
2. Adding a large perturbation along input directions that the base model’s activations rarely occupy, which changes the update but barely changes the layer’s output on real inputs;
3. Training a freshly initialized adapter to reproduce the outputs of the original;
4. Merging with task-trained adapters;
5. Continued fine-tuning;
6. Pruning;
7. A false claim, in which a claimant registers the average of many public adapters and hopes that adapters it did not train will pass.

The third strategy differs from the others. It does not edit the original weights but re-derives an adapter from the original’s behavior, which is close to training a new adapter for the same task. Section 6.6 shows that strategies 1 to 3 remove the fingerprint at a small cost in utility. They aim at removing the fingerprint, and Section 2.1 places them outside the threat model; for strategy 2, Section 6.6 also evaluates optional data-aware probes. An adversary who can query the verifier many times could also learn about the key from the verdicts, and Appendix B describes the limits a registry places on such queries.

### 5.6. Interpreting an Accepted Match

It remains to say precisely what an accepted verdict means since the guarantees above are narrower than the word ownership suggests. An accepted bundle has a deliberately narrow interpretation: the factors decoded from the custodian’s named file meet a threshold that appeared in an earlier authenticated record. The result does not reveal the path by which that file reached the custodian. It can support a broader provenance investigation, but only as technical evidence about this file-policy pair.

This distinction is apparent under an equivalent refactorization. Let *Q* be invertible. The stored factors BQ and $Q^{-1}A$ change, as do their commitments, while their product remains $\left(BQ\right)\left(Q^{-1}A\right)=BA$. In exact arithmetic, they therefore give the same directional responses in Equation (2), and the balanced form of Section 4.4 maps both files to the same encoded factors. ZK-AdapterMark does not require the candidate commitment to equal the original commitment; it authenticates the new file and proves that the resulting update passes the registered test.

## 6. Experimental Evaluation

The construction makes claims that only measurement can settle. The circuit must stay small once the commitments are added, and the fingerprint must survive ordinary edits while rejecting adapters unrelated to the registered one. We first measure the cost of the circuit and of a complete dispute. We then apply common edits to 148 real adapters, compare 904 adapters trained independently, test the fair-bit model, and run seven adaptive strategies. The section ends with the choice of the policy parameters, the setup artifacts, and a comparison with existing systems.

Unless stated otherwise, a layer fingerprint has m=128 response bits and a layer passes at τ=96 agreements. The threshold was derived in Section 5. For an adapter unrelated to the registered one, the agreement count is binomial with mean 64 and standard deviation about 5.7. The value τ=96 lies 5.7 standard deviations above that mean, a false-accept probability of about $6\times 10^{-9}$ per layer. Setting a bit-agreement threshold from this binomial false-positive analysis is the standard practice in watermark and fingerprint detection [4,19]. Related adapters, by contrast, retain most of their bits after ordinary edits, as Section 6.3 shows; so, three quarters of the length leaves margin on both sides. Section 6.4 measures that margin directly and Section 6.5 checks the model against the data. Five passing layers among the six registered layers are required for an adapter decision. Proofs were generated with Groth16 [16] from circuits written in Circom 2.2.3 [15]. Every constraint count, time, memory figure, key size, and proof size in this section comes from the complete circuit of Section 4.3. All of them were measured on one Alibaba Cloud server with 16 vCPUs and 64 GiB of memory, and the prover used 16 threads. Appendix A describes the circuit version, the tools, and the timing procedure. The timed circuit took the low 253 bits of each Poseidon output. Appendix A also describes the change to 252 bits, which leaves every constraint count unchanged.

### 6.1. Constraint Cost of the Factor-Aware Circuit

**Figure 3:**
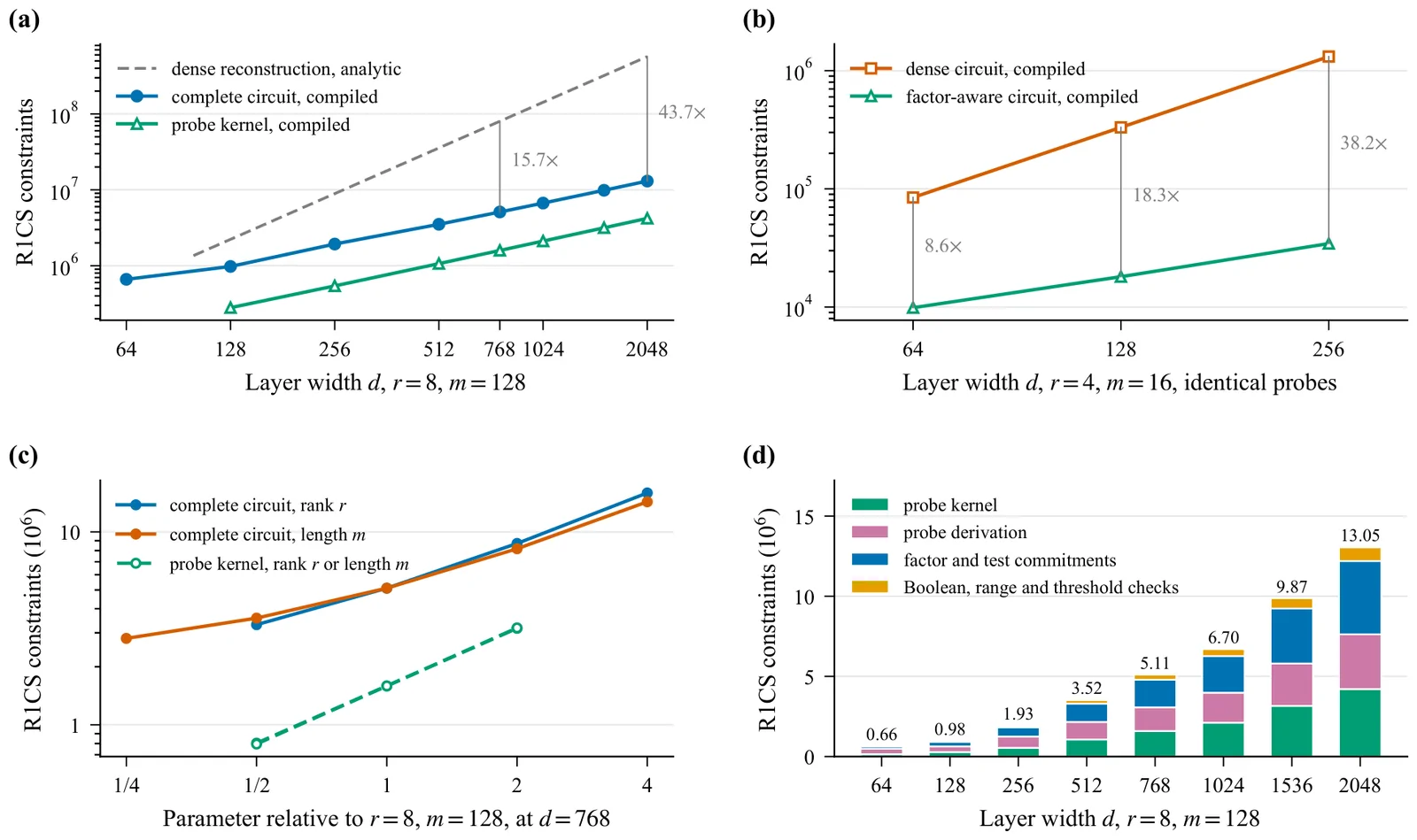
Constraint cost of the directional fingerprint. (**a**) Compiled counts of the probe kernel and the complete per-layer circuit against layer width at r=8 and m=128, with the analytic dense estimate for comparison. (**b**) Dense and factor-aware circuits compiled for identical probes at r=4 and m=16. (**c**) Kernel and complete-circuit cost at width 768 when the rank or the fingerprint length ranges from a quarter to four times its default. (**d**) Decomposition of the complete circuit into probe kernel, probe derivation, commitments, and the Boolean, range, and threshold checks, using compiled counts for each building block; the number above each bar is the compiled total in millions. R1CS: rank-1 constraint system.

We began with a controlled comparison of the probe evaluation part of the circuit alone, which we call the *kernel*, with only the evaluation route changed. Both kernels used rank 4 and 16 probes. One explicitly produced BA; the other used Equation (5). At width 128, compilation gave 331,350 constraints for the dense version and 18,070 for the factor version. Thus, the 18.34× reduction in Figure 3b is measured for the same directional scores rather than inferred from asymptotic notation. The same panel shows the ratio growing from 8.6× at width 64 to 38.2× at width 256, beyond which the dense circuit became too large to compile on the client.

The kernel omits what the complete circuit must also do: open the two commitments, derive the probe directions from the key, and apply the threshold. Table 2 and Figure 3a therefore give a second accounting of the complete circuit at deployment parameters. These additions raise the kernel counts by factors of 3.21 and 3.10 at widths 768 and 2048, respectively. That overhead is sizeable. Even after it is included, however, the complete circuits remain 15.7× and 43.7× smaller than analytic dense reconstruction. The widening gap follows from the different growth terms. Dense reconstruction depends on $d_{\mathrm{out}}d_{\mathrm{in}}$, while the factor path is linear in the layer dimensions for fixed rank and fingerprint length, as the kernel counts compiled at seven widths confirm. Figure 3d separates the complete circuit into kernel, probe derivation, commitments, and checks using compiled counts for each building block; at every width their sum equals the compiled total. Figure 3c shows that at width 768, doubling the rank from its default raises the cost by a factor of 1.71, and doubling the fingerprint length raises it by 1.60. Further doublings approach a factor of two because the part that does not depend on the doubled parameter takes a smaller share. The deployment-scale result therefore retains the saving observed in the controlled kernel.

### 6.2. Proving and Verification Cost

Table 3 gives the cost of the complete circuit at four widths, measured as described in Appendix A. Constraint counts translate into time very differently for the prover and for the verifier. From width 768 to 2048, the constraint count grows by a factor of 2.55, the proving time by a factor of 2.26, and the peak memory of the setup from 15.9 to 36.6 GiB. The cost of the verifier hardly changes: native verification takes about 3.8 ms per proof, resident verification in snarkjs about 6.6 ms, and every proof has about 0.7 kB. A wider layer therefore burdens mainly the proving service. The timed circuit enforces the complete per-layer relation. The verifier checks the signatures, the context equality, the policy range, and the adapter-level rule outside it, all on public values.

The setup is paid once per shape. At width 768, the initial circuit-specific key takes 443 s with a peak of 15.9 GiB, and one phase 2 contribution another 245 s. The proving key has 2.49 GB, about 487 bytes per constraint, and the verification key has 3.8 kB at every width. The data-aware variant of Appendix C has 5.4 percent fewer constraints at this width and proves in 9.60 s.

For an adapter-level run, we used the six attention layers of a public DistilBERT text-classification adapter. Its factors have rank 4, and padding them with zeros to rank 8 does not change BA. Table 4 divides the cost of registration and dispute among the roles. At registration, preparing the inputs and witnesses of the six layers takes 20 s, the six registration proofs take 58.7 s, and the registry checks them in 0.24 s. In a dispute, the custodian reads the 2.5 MB file, computes its digest, encodes the factors, and commits to them in about 2.0 s. The prover prepares the witnesses in 20 s and produces the six proofs in 58.7 s sequentially, 56.0 s with two concurrent jobs, and 55.6 s with six, at a peak of 3.6 GiB. The native prover already uses all 16 threads; so, concurrency gains little. The verifier checks the records in 2.0 ms and the six proofs in 43 ms with a resident snarkjs verifier, including the reading of the proof files, and the five-of-six rule accepts the adapter. Run one after another, the steps of the dispute in Table 4 take about 81 s, most of it for the six proofs.

A hub service is more likely to verify from JavaScript; our measurements use snarkjs 0.7.6. Starting a new snarkjs process for each proof gives a median of 0.31 s, while keeping one verifier process resident brings it to 6.6 ms, about 47 times less. Most of the fresh-process latency is initialization; so, a continuously operating hub should keep the verifier resident.

### 6.3. Fingerprint Persistence Under Transformation

**Figure 4:**
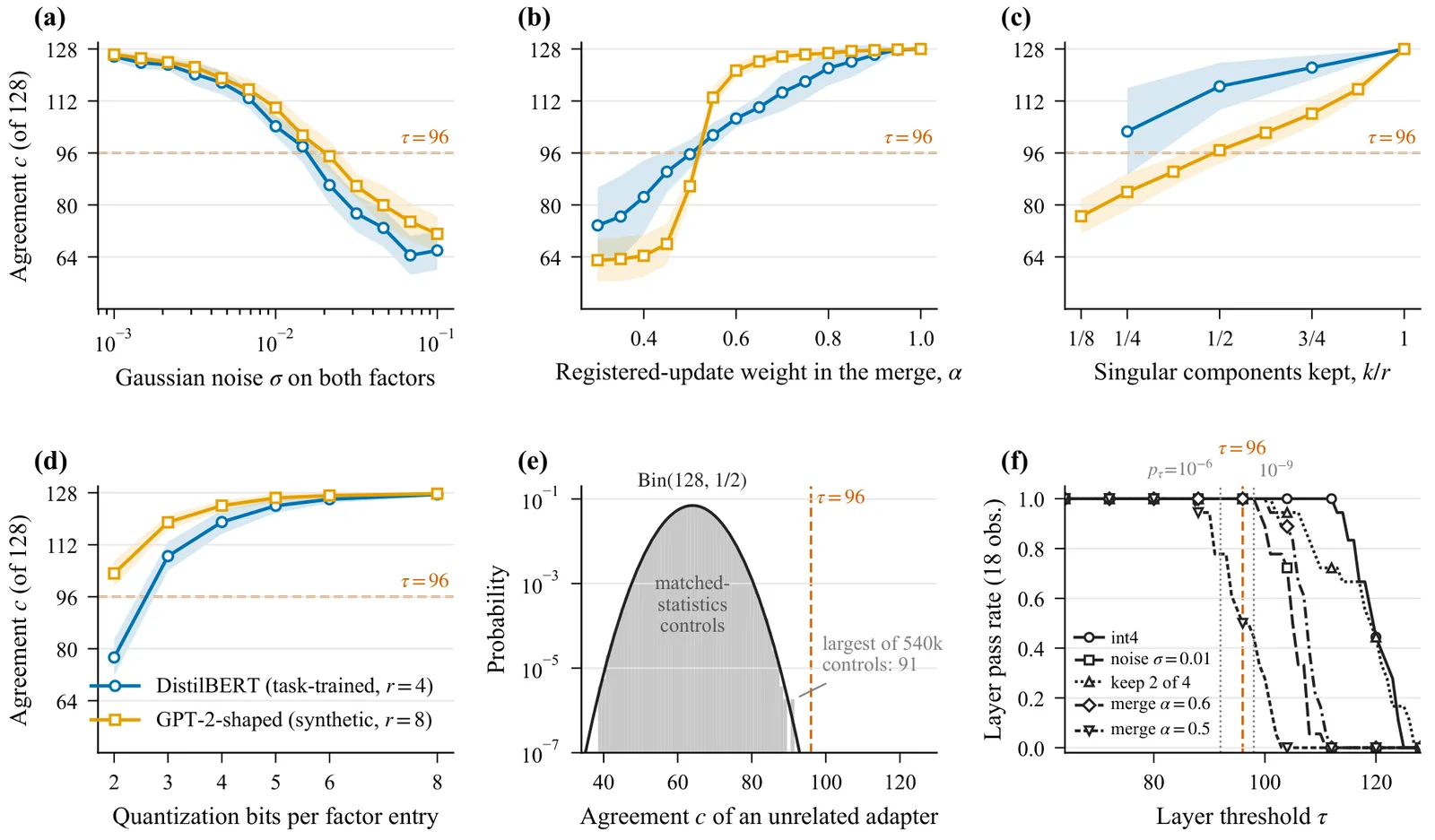
Fingerprint agreement under transformation sweeps and for unrelated controls. (**a**–**d**) Mean agreement and one standard deviation across 18 DistilBERT and 36 GPT-2-shaped layer-key observations as a function of noise level, merge weight, retained singular components, and quantization bits; the dashed line is the layer threshold τ=96. (**e**) Distribution of agreement counts for 540,000 matched-statistics unrelated controls against the Bin(128,1/2) mass function, on a logarithmic scale. (**f**) Fraction of DistilBERT observations passing as a function of the layer threshold for five transformed conditions; the grey lines mark the thresholds at which the per-layer false-accept probability of an unrelated adapter falls below $10^{-6}$ and $10^{-9}$.

The cost results say nothing about whether the fingerprint is secure. We therefore tested the fingerprint itself in two respects: whether it still recognizes the registered adapter after the edits a copier would make, and whether it wrongly accepts adapters that are unrelated. For the first question, we altered a task-trained DistilBERT adapter whose six query-projection updates are 768×768 and rank 4. Three keys were evaluated on every layer, yielding 18 key-layer observations for each setting. A second, synthetic adapter with the rectangular geometry of a GPT-2 attention projection, $d_{\mathrm{out}}=2304$ and $d_{\mathrm{in}}=768$ at rank 8, has twelve layers and three keys and provides 36 observations per setting. Quantization was performed symmetrically and separately for each factor tensor at 2 to 8 bits. For the noise cases, independent Gaussian samples were added to both factors at thirteen log-spaced standard deviations between $10^{-3}$ and $10^{-1}$. Rank reduction operated on BA: we kept its largest singular components and refactorized the truncated matrix at the original rank. The merge cases mixed the original update with a synthetic update, a random update with the same factor statistics, at weights from 0.30 to 1.00 in steps of 0.05. The mixture was then refactorized at rank *r* by truncated singular-value decomposition. These truncations produce the candidate file, which stores the truncated factors at the original rank, and the custodian only decodes that file. Figure 4a–d shows the full sweeps, and Table 5 lists the DistilBERT settings discussed in the text.

The results appear in Table 5 and Figure 4. Every DistilBERT layer passes after quantization to 8, 4, or 3 bits, after removal of one or two of the four singular components, under noise up to σ=0.01, and for merges retaining at least 0.7 of the original update. In all of these cases, the five-of-six rule accepts the adapter under each of the three keys. The 8-bit result is nearly unchanged, with a mean loss of 0.5 agreement among 128 probes. Boundary cases are more revealing. At σ≈0.015, thirteen of the eighteen observations still pass, but only one key retains five passing layers; at σ≈0.022, a single observation passes. Keeping only one of the four components spreads the counts from 79 to 125, with twelve passing observations and one accepting key. Two-bit quantization fails in every layer.

The merge sweep locates the dilution boundary. At weight 0.55, all but one observation pass and every adapter decision accepts. At equal weight the average count, 95.6, lies almost on the layer threshold; nine observations pass, but no key reaches five passing layers; so, the adapter is rejected. At weight 0.45 five observations pass. The transition is sharp because the rank-*r* truncation that follows a merge keeps mostly the components of whichever update carries more weight; so, the fingerprint is either largely preserved or largely discarded. These coefficients are not claimed as universal cutoffs; they belong to this adapter, these keys, and the chosen threshold. Whether the rejected half-strength component remains useful for the original task is a separate empirical question, taken up in Appendix B.1, and should be part of deployment calibration.

The GPT-2-shaped layers give the same ordering, and Table A3 in Appendix E lists their counts. One difference matters for deployment. Removing four of eight components passes only 23 of 36 observations, whereas the rank 4 adapter passed the removal of two components in every layer. A tolerance inferred at one rank should therefore not be transferred unchanged to adapters that spread their update over more components.

These two adapters cannot show that the results are general, and the factors of the second are synthetic. We therefore applied the same transformations to 148 real adapters. They come from 17 base models in ten families: DistilBERT, BERT, RoBERTa, GPT-2, OPT, Phi, Gemma, Llama, Qwen2.5, and Mistral-7B. Their ranks range from 4 to 256 and their layer shapes from 768×768 to 4096×4096, including the 2304×768 projection of GPT-2. We trained 33 of them; the others come from Hugging Face, LoRA Land, and Lots-of-LoRAs.

**Figure 5:**
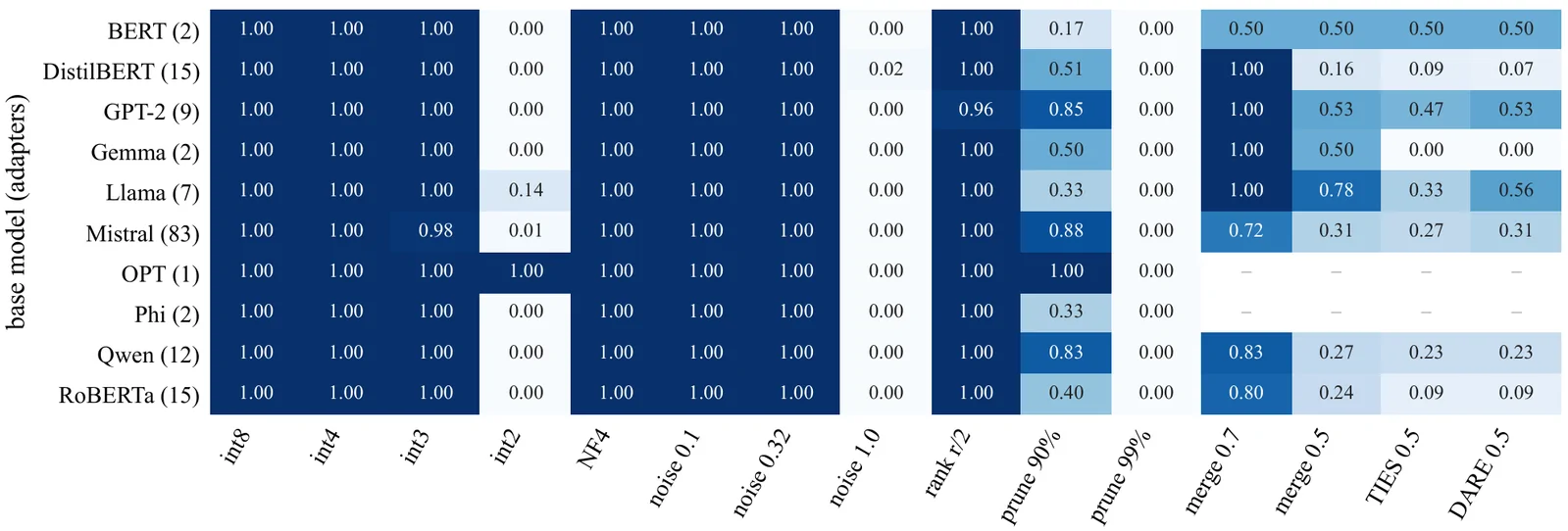
Adapter-level acceptance of the real adapters under each transformation, grouped by base-model family with the number of adapters in parentheses; an adapter is accepted when at least five of six layers pass. The merge partner is another adapter of the same base model; OPT and Phi had none. Each cell gives the acceptance rate; darker blue marks a higher rate, and a dash marks a merge for which no partner was available. The labels int8 to int2 denote quantization to 8 to 2 bits, NF4 the 4-bit NormalFloat format, and TIES and DARE two merging methods.

Figure 5 gives the adapter-level acceptance per model family. Every family is accepted after quantization to three or more bits, after 4-bit NormalFloat (NF4) quantization, and under noise of relative strength up to 0.32, except Mistral at 0.98 for three bits. After halving the rank only GPT-2 falls below 1.00, to 0.96. Two-bit quantization removes the fingerprint in every family except OPT, Llama at 0.14, and Mistral at 0.01. Noise of relative strength 1.0 and 99 percent pruning remove it in every family, apart from 0.02 for DistilBERT under noise. Pruning to 90 percent and merging lie at the boundary, with results that differ between families.

**Figure 6:**
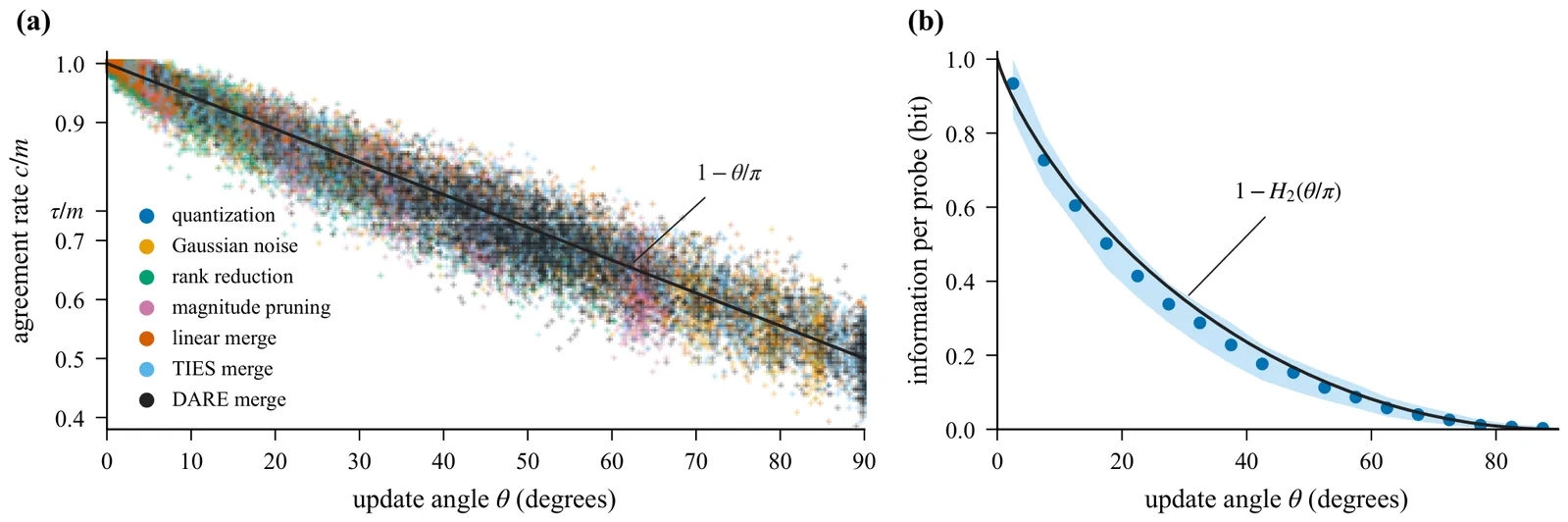
Agreement and update angle. (**a**) Agreement rate c/m of every transformed layer against the angle between the original and the transformed update, coloured by transformation; the black line is 1−θ/π, and the threshold τ/m is marked on the vertical axis. (**b**) Plug-in information per probe, $1-H_{2}\left(1-c/m\right)$: points are medians in 5-degree bins, the band is the interquartile range, and the black line is $1-H_{2}\left(\theta /\pi \right)$.

The tolerated strength of a transformation differs between adapters, but the difference is explained mainly by how far the update turns. Figure 6a plots the agreement rate of every transformed layer against the update angle, and the points lie close to the law 1−θ/π of Proposition 3. The relative noise level at which half of the layers of an adapter are rejected ranges from 0.56 to 1.07, nearly a factor of two. The angle at that point lies between 39.7° and 44.5° for the middle half of the adapters, close to the 45° of the analysis. Figure 6b restates the same data as plug-in information per probe.

One public adapter also showed why the screening step at registration is needed. In an OPT-350m adapter, the up-projection factor is zero in all 48 adapted layers; so, BA=0 and every directional measurement is zero. A sign string from such a layer carries no identifying information and would be easy for arbitrary candidates to reproduce. The claimant’s screening step of Section 2 excludes these layers before registration. Degenerate layers are thus a real occurrence in public repositories, not only a pathological construction.

### 6.4. Separation from Unrelated Adapters

**Figure 7:**
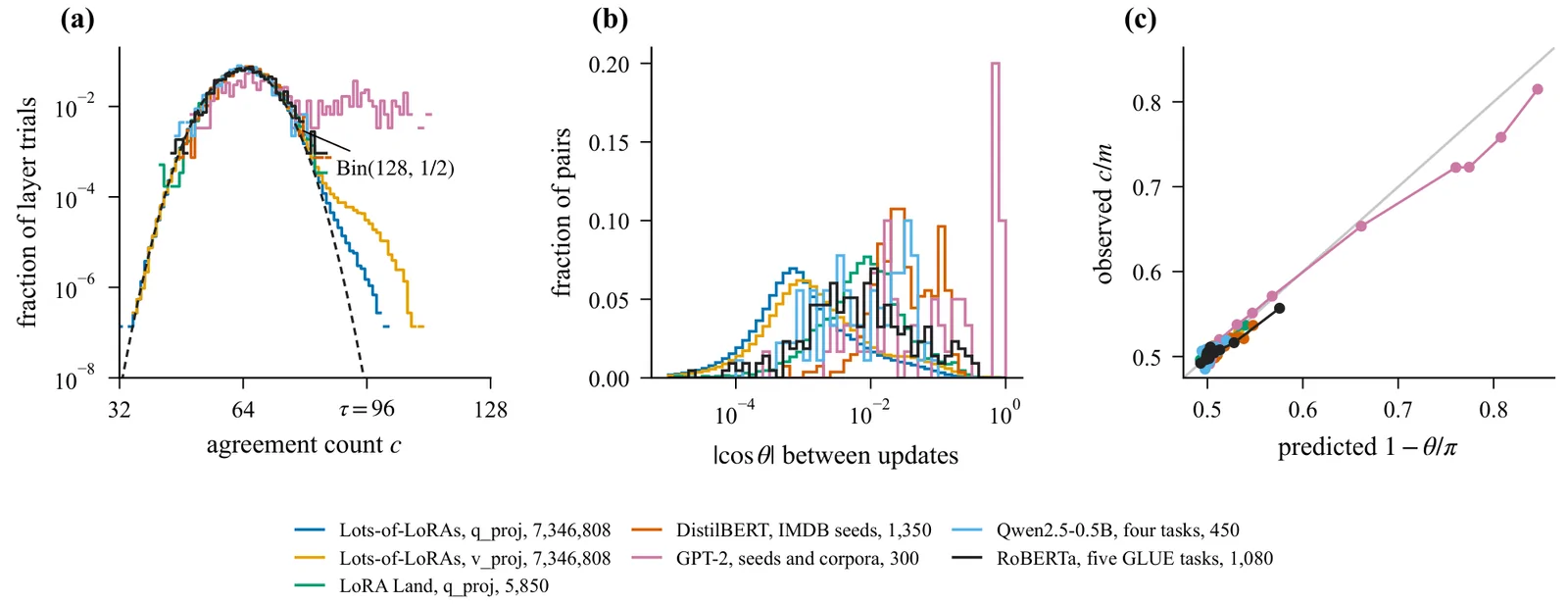
Independently trained adapters. (**a**) Frequency of the layer-level agreement count in each collection against Bin(128,1/2); the numbers in the legend are layer-level comparisons. (**b**) Absolute cosine between the updates of each pair of adapters, per layer. (**c**) Agreement rate predicted from the angle, 1−θ/π, against the observed rate, averaged in bins of the prediction; the grey line marks equality. Colors identify the collections listed below the panels; in (**a**), the dashed curve is the binomial distribution, and in (**c**), each point is one bin.

Persistence is only half of the requirement. If an adapter with no relation to the registered one could also pass the registered test, a match would prove nothing. Random factors with the statistics of the registered adapter are one kind of control. Independently trained adapters are harder to separate, and we use them as the main controls. The largest collection comes from Lots-of-LoRAs and holds 904 adapters trained from the same base model, Mistral-7B-Instruct-v0.2, on different Super-NaturalInstructions (SuperNI) tasks with the same rank and target modules. Each unordered pair of adapters was compared on six q_proj layers under three keys. The probes do not depend on which adapter of a pair is registered; so, one comparison covers both directions. This gives 7,346,808 layer-level and 1,224,468 adapter-level comparisons. The agreement counts have mean 64.2 and standard deviation 5.70, close to the values 64 and 5.66 of Bin(128,1/2). Figure 7a shows that their frequencies follow the binomial down to about $10^{-6}$, with a tail only at the far right.

The tail comes from pairs of tasks whose updates are similar. Of the layer-level comparisons, 27 reach the threshold of 96, and in each of them the cosine between the two updates is at least 0.34, with a median of 0.56. Among comparisons whose cosine is below 0.1 in absolute value, the largest count is 92 and none reaches the threshold. This agrees with Proposition 3, since in this collection, the agreement rate follows the angle between the updates closely. In Figure 7c, the rate predicted from the angle differs from the observed rate by 0.02 on average. A layer that passes therefore indicates that the two updates are similar. It does not indicate a failure of the bit statistics. At the adapter level, no pair passes more than one layer under any key, while the rule requires five; so, none of the 1,224,468 adapter-level comparisons is accepted.

The other collections lead to the same conclusion. The tail of the v_proj layers is heavier, with 946 layer-level comparisons at the threshold, but no pair passes more than three layers, and no adapter-level comparison is accepted. The 26 adapters of LoRA Land, trained by another organization, give 5850 layer-level comparisons with a largest count of 85. Adapters trained with the same recipe on the same data and differing only in the random seed are the hardest case. Ten DistilBERT seeds have cosines of at most 0.21 and counts of at most 86, and nine RoBERTa adapters on five General Language Understanding Evaluation (GLUE) tasks reach at most 85. Six Qwen2.5-0.5B adapters on four tasks, three of them Alpaca seeds, reach at most 82. Three GPT-2 seeds trained on the same corpus have cosines between 0.64 and 0.89; so, some of their layers pass, but no pair passes more than four layers and no adapter is accepted. Table 6 summarizes the collections.

The matched-statistics controls are listed in Table 6. Their 540,000 counts have mean 64.0 and standard deviation 5.65, and Figure 4e shows that their frequencies follow the binomial down to about $10^{-6}$.

Two kinds of numbers should be kept apart here. The sample observes no false accept among 540,000 independently generated controls, and this observation alone bounds the per-layer false-accept probability of such controls by $3/540,000\approx 5.6\times 10^{-6}$ at 95% confidence [20]. The far smaller values of Section 5.4, $6.4\times 10^{-9}$ per layer and the adapter-level estimate, are predictions of the binomial model, which the sample supports but cannot observe directly. No comparison between independently trained adapters was accepted either. These comparisons reuse the same adapters and keys; so, they are not independent trials, and we do not convert their number into a confidence bound.

Figure 4f shows what the threshold trades off. For five DistilBERT conditions, the fraction of passing observations is plotted against τ, together with the thresholds at which the binomial false-accept probability of one unrelated layer falls below $10^{-6}$, at 92, and below $10^{-9}$, at 98. Raising the threshold to 106 would still accept 4-bit quantization in every layer but would reject most layers at σ=0.01. Lowering it to 88 would accept almost every observation of the equal-weight merge, whose counts range from 87 to 102, at a per-layer false-accept probability near $10^{-5}$. The operating point τ=96 accepts the ordinary transformations with some margin and leaves the diluted merge on the rejecting side.

### 6.5. Validation of the Fair-Bit Model

**Figure 8:**
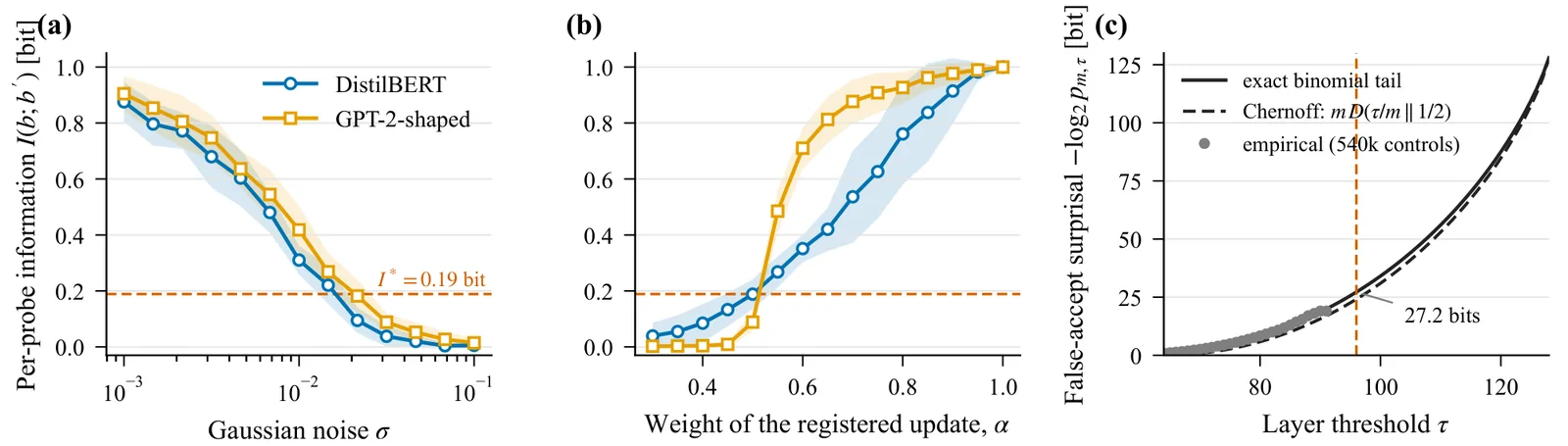
Information-theoretic view of the measurements in Section 6. (**a**,**b**) Plug-in per-probe information $1-H_{2}\left(\varepsilon \right)$ between registered and candidate responses, with ε the observed fraction of disagreeing probes, along the noise and merge sweeps, with one standard deviation across observations; the dashed line is the value $I^{*}$ implied by τ=96. (**c**) Surprisal of a false accept, $-\log_{2}p_{m,\tau }$, from the exact binomial tail, the Chernoff bound mD(τ/m∥1/2), and the empirical control sample.

The threshold analysis of Section 5 rests on the fair-bit model; so, we checked the model against the data in three ways. First, for the matched-statistics controls, the observed mean, standard deviation, and tail frequencies down to about $10^{-6}$ agree with the binomial prediction, as Figure 4e showed. Figure 8c plots the same comparison in bits, next to the exact tail of Equation (9) and the Chernoff bound of Equation (11). Even a sample of 540,000 controls cannot observe the event at τ=96 directly; so, the far-tail value quoted in Section 5 is a model prediction that the sample supports but does not observe. Second, Figure 8a,b plots the plug-in per-probe information of Equation (10), with ε in place of η, along the noise and merge sweeps. The information decays gradually with noise but drops almost as a step across the equal-weight merge. This restates in bits what the agreement curves of Figure 4 showed in counts, and the information threshold $I^{*}$ separates the two regimes at exactly the settings where the agreement curves cross τ.

A third test uses the 904 independently trained Lots-of-LoRAs adapters and checks the parts of the fair-bit model separately. Bit bias exists in this population, mainly in later layers, where adapters of one base model share a common component of their updates. For a fixed pair of adapters, the agreement counts over 1000 keys follow the binomial law, and the counts of different layers are only weakly correlated. Inserting the measured distribution of *p* into Equation (13) gives a plug-in estimate of $4.8\times 10^{-15}$ for the adapter-level false-accept probability, against $6.5\times 10^{-41}$ under fair bits. This value describes the measured collection under the model and does not bound the false-accept probability of adapters outside it. Appendix E reports the tests in detail.

### 6.6. Adaptive Attacks

**Figure 9:**
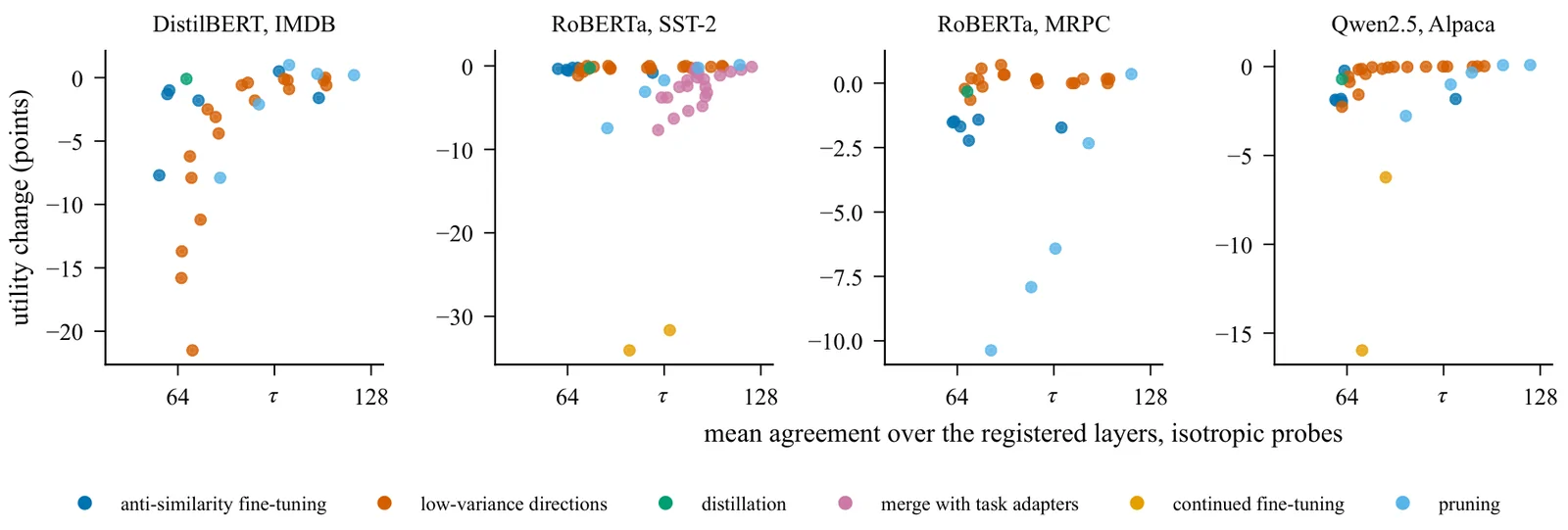
The first six strategies of Section 5.5 on the four registered adapters, isotropic probes. The horizontal axis is the mean agreement over the six registered layers, with the layer threshold τ marked. The vertical axis is the change in utility relative to the registered adapter: accuracy points for the classifiers, and for Qwen2.5-0.5B the relative change in perplexity in percent, with the sign reversed so that lower values are worse. The false claim is reported in the text.

We measured the seven strategies of Section 5.5 on four registered adapters: DistilBERT for IMDB, RoBERTa for SST-2 and MRPC, and Qwen2.5-0.5B for Alpaca. Six layers of each adapter are registered, and the adapter is accepted when at least five of them pass. Each attack records the task utility, accuracy for the classifiers and perplexity for Qwen, together with the agreement of every registered layer. Figure 9 shows all results.

Pruning, merging, and continued training change the update, and the outcome follows the angle as Proposition 3 predicts. Layers keep passing, while their angle stays well below 45°. At 80 percent sparsity all six layers pass on all four adapters, and at 90 percent the DistilBERT and Qwen adapters are still accepted. At 99 percent the fingerprint is lost, while the accuracy of the classifiers falls by 7.5 to 10.4 points. The RoBERTa adapter for SST-2 was also merged with the adapters of four other GLUE tasks. After linear and DARE (drop and rescale) merging, all six layers pass as long as the registered adapter keeps a weight of at least 0.4. TIES (trim, elect sign, and merge) merging needs a weight of at least 0.5. After 250 steps of continued training on the same task, all six layers pass on all four adapters. After 1000 steps, five layers still pass on Qwen, while the updates of the two RoBERTa adapters have turned by 32° to 47° and two and four layers pass.

The three strategies that aim at the fingerprint remove it at a small cost in utility. Perturbing the factors along input directions of low variance with twice the norm of the factor leaves at most two passing layers on the three classifiers and changes their accuracy by at most 1.8 points. On Qwen, a perturbation of once the norm already leaves at most three layers, with unchanged perplexity. Continued training against the squared cosine with a penalty weight of 10 turns every registered layer beyond 87°. It costs 0.2 to 1.7 points of accuracy and raises the perplexity of Qwen by 1.9 percent. Distillation into a freshly initialized adapter leaves every layer beyond 73°, at an accuracy loss of at most 0.3 points and a perplexity increase of 0.7 percent. These three strategies lie outside the scope set in Section 2.1. The false claim, in contrast, fails: after the claimant registers the average update of 10 to 200 public adapters, none of these adapters and none of the held-out ones is judged to derive from it.

**Figure A1:**
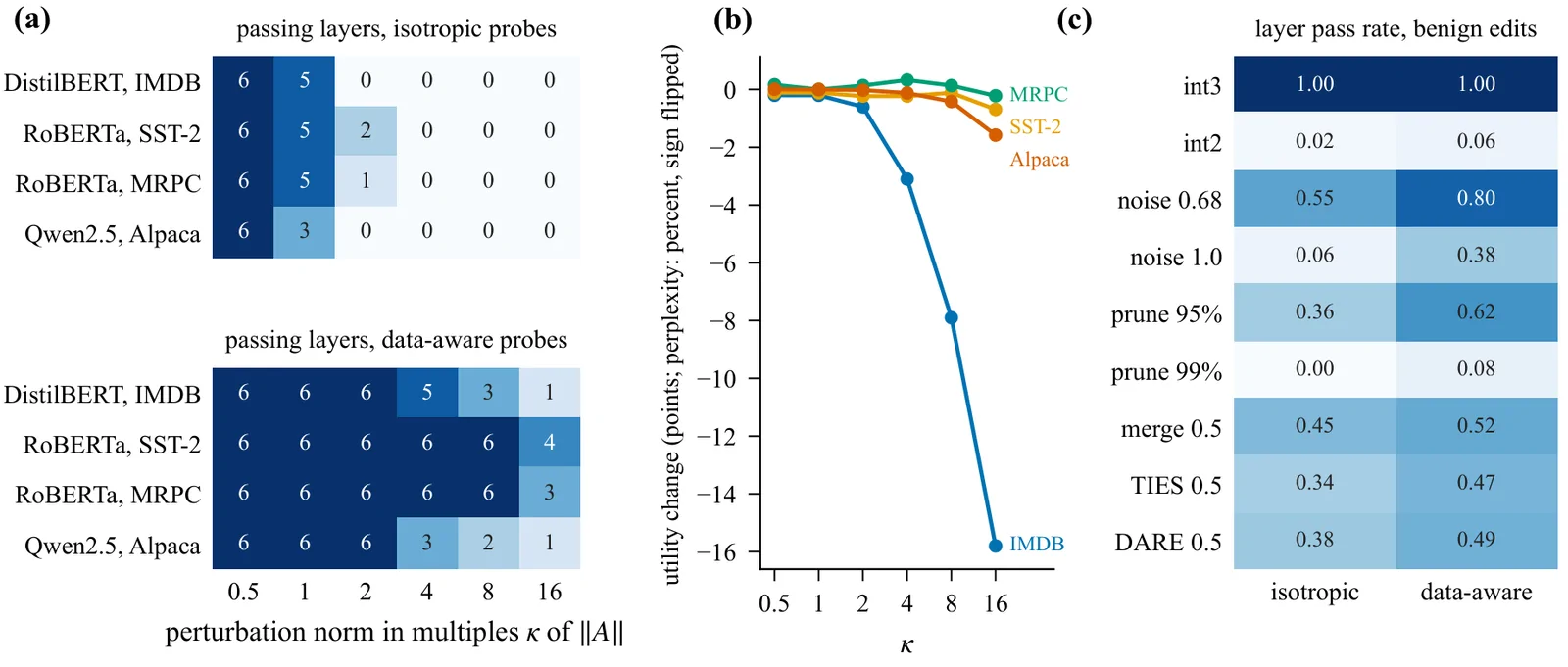
Isotropic and data-aware probes under the low-variance perturbation. (**a**) Passing registered layers of each adapter when the perturbation norm is κ times the norm of the factor, median over three perturbations. (**b**) Utility change under the same perturbations, in the units of Figure 9. (**c**) Layer pass rate of both kinds of probes under the benign edits of Section 6.3.

The low-variance perturbation exploits a property of the isotropic probes: their input directions are spread evenly and therefore also cover input directions that the model hardly uses. Appendix C describes optional data-aware probes, which restrict the input directions to the principal input subspace of the base model. They raise the perturbation needed to reverse the decision by a factor of four on DistilBERT and Qwen and eight on the two RoBERTa adapters. As in Figure A1, the decision is judged by the median of three perturbations. On DistilBERT, one of the three perturbations at four times the norm already reverses the data-aware decision, at a cost of 4.4 points of accuracy. Data-aware probes are also more stable under pruning: at 95-percent sparsity three of the four adapters are still accepted, against one with isotropic probes. They do not stop the other two strategies, which retrain an update with a different direction. We do not make them the default because they also raise the agreement between adapters trained independently with the same data and recipe. Among three GPT-2 seeds trained on WikiText-103, every pair passes all six layers under every key with data-aware probes, and at most four with isotropic probes. A claimant whose training data are not public may choose data-aware probes.

### 6.7. Sensitivity of the Acceptance Policy

**Figure 10:**
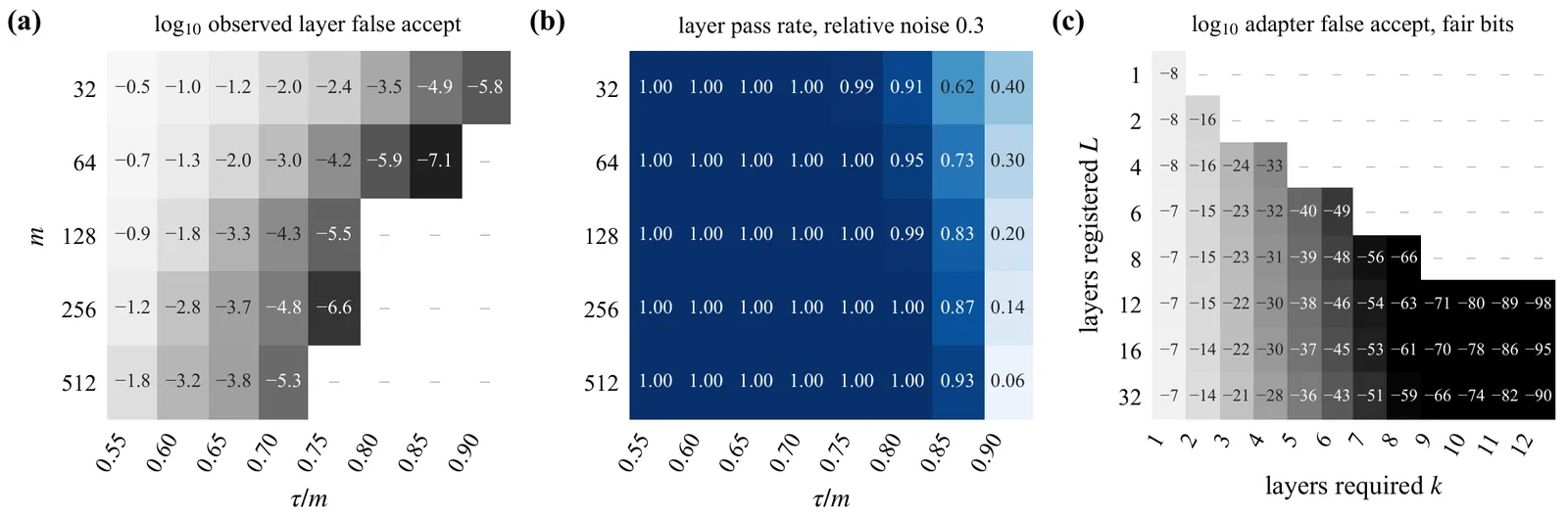
Sensitivity of the acceptance policy on the 904 Lots-of-LoRAs adapters. (**a**) Observed layer-level false-accept rate, $\log_{10}$; a dash marks no false accept among 26,121,984 layer-level comparisons. (**b**) Layer pass rate under noise of relative strength 0.3. (**c**) Adapter-level false-accept probability under the fair-bit model for m=128 and τ=96 when at least *k* of *L* layers must pass. Darker gray marks a lower false-accept rate in (**a**) and (**c**), and darker blue marks a higher pass rate in (**b**); in (**c**), a dash marks a combination that requires more layers than are registered.

We varied the parameters of the acceptance policy on all 32 q_proj layers of the 904 Lots-of-LoRAs adapters. The fingerprint length *m* ranged from 32 to 512 and the threshold ratio τ/m from 0.55 to 0.90. The number *L* of registered layers ranged from 1 to 32, together with the number *k* of layers the rule requires and the way the layers are selected. The selections were evenly spaced layers, the first, the middle, or the last layers, a random choice, and the layers with the largest mean update norm in the collection. Because the probes are independent, one run with 512 probes yields the results for every shorter fingerprint. The negatives are all pairs of adapters under two keys, 26,121,984 layer-level comparisons, and persistence uses 30 of the adapters under representative transformations. Figure 10 shows the main results.

A longer fingerprint improves both sides. At τ/m=0.75, raising *m* from 64 to 512 lowers the observed layer-level false-accept rate from $6.4\times 10^{-5}$ to zero and raises the pass rate after 90 percent pruning from 0.87 to 0.98. The pass rate after an equal-weight merge stays between 0.46 and 0.51 for every *m* so that merge remains the boundary case. Merging with an unrelated update at equal weight turns the update by about 45°. Raising τ/m instead trades persistence for fewer false accepts. At m=128, a ratio of 0.80 instead of 0.75 removes every observed false accept, while the pass rate under noise of relative strength 0.3 falls from 1.00 to 0.99, and to 0.83 at 0.85.

The adapter-level rule decides how low the false-accept rate can go. With at least three passing layers required, no adapter-level false accept is observed for any *L* or selection strategy at m=128 and τ=96. With two layers the rate is at most $3.7\times 10^{-6}$, and with one layer it ranges from 0 to $9.8\times 10^{-5}$ depending on which layers are selected. The fair-bit model gives far smaller values, $6.5\times 10^{-41}$ for the default five of six layers in Figure 10c. These values are predictions of the model and were not observed.

The cost grows linearly in *m* and *L*. At width 768 and rank 8, the complete circuit has 3.57, 5.11, 8.18, and 14.33 million constraints for *m* of 64, 128, 256, and 512, and every additional registered layer adds one proof. The default operating point, m=128, τ=96, and five of six layers, leaves margin on both sides. Where a lower false-accept rate is needed, m=256 provides it at 1.6 times the constraint count.

### 6.8. Setup Artifacts and Proof Systems

Groth16 needs a separate setup for every circuit shape, and a shape is fixed by the layer dimensions, the rank, the fingerprint length, and the circuit version. A hub that accepts adapters for many base models therefore has to decide how many shapes to support and who holds their keys. Three properties keep the number small. First, the adapters of one base model target a handful of projection matrices, and models of one family share these shapes. Second, ranks can be grouped. Padding *B* with zero columns and *A* with zero rows up to the next supported rank leaves BA, and hence every response, unchanged, at the cost of proving a larger circuit. Third, the fingerprint length is a policy constant fixed by the version; so, it does not vary between adapters.

The context of a layer records the rank of its circuit. A candidate file of lower rank is padded with zeros to that rank, which leaves its update unchanged. A candidate of higher rank does not fit the circuit; so, a dispute cannot establish a match under that policy. The parameter path in the context fixes which module of the file is tested, and a missing module or a module of another shape yields no valid proof.

Each shape produces two artifacts with different owners. The proving key grows linearly with the number of constraints and is needed only by the parties that prove, the claimant at registration and the prover in a dispute. The verification key has a constant size of a few kilobytes, and it is the only artifact the hub stores. Neither key travels with the adapter. Users download only the adapter file, and a proving key moves once per shape to each party that proves. The setup itself has two phases. The first is a universal powers-of-tau file, which we take from an existing public ceremony. The second is specific to the circuit and must be run once per shape, preferably with several independent contributors [21].

We counted the circuit shapes needed for the 27 base models with the most adapters on Hugging Face. Layer shapes come from the configuration files of these models and ranks from their adapters. Up to 60 adapters were sampled per base model, and ranks were padded to 8, 16, 32, 64, or 128, or to the next power of two above that. The 3501 sampled adapter modules map to 179 shapes. Ordered by frequency, the first 20 shapes cover 44 percent of the modules and the first 80 cover 87 percent, as Table A4 in Appendix E shows. For shapes that were not compiled, the constraint count is estimated with the fitted model of Appendix A.

The verifier holds only verification keys, about 3.8 kB per shape and less than 1 MB for all 179. The proving keys are held by the parties that prove and grow with the constraint count, at about 487 bytes per constraint as measured at widths 64 and 768. At width 2048, the measured value is 463 bytes per constraint; so, the estimate is slightly conservative for larger shapes. The first 20 shapes need about 261 GB, and the 131 shapes of rank at most 64, which cover 94 percent of the modules, need about 5.0 TB. All 179 shapes need 20.7 TB, most of it for a few adapters of rank 256 or more and for multilayer perceptron (MLP) projections such as 18,944 × 3584. Because registration also needs a proof, the key of a shape must exist before the first adapter of that shape is registered. Shapes that no claimant registers need no key since each shape needs only one circuit-specific contribution on top of the public powers-of-tau file.

Proof systems with a universal setup, such as PLONK [22] and FFLONK [23], remove the second phase. One structured reference string then serves every shape up to a maximum size, and a new shape requires only a public preprocessing step. We measured PLONK and FFLONK on the complete circuit at width 64, with 662,302 constraints, and Table A5 in Appendix E lists the results. To compare the systems under one prover, all three were proved with snarkjs. One proof takes 11.8 s with Groth16, 657 s with PLONK, and 826 s with FFLONK, and verification in a new process takes about 0.3 s for each. The proofs have 706, 2252, and 2259 bytes, and the proving keys 0.32, 4.89, and 6.50 GB. A universal setup thus removes the circuit-specific contribution per shape. In this measurement, however, its proofs are 56 to 70 times slower, and its proving keys are 15 to 20 times larger. Its peak proving memory is 16.6 to 31.5 GiB, against 5.5 GiB for Groth16. FFLONK already needs 31.5 GiB to prove at this width; so, larger widths were not measured. With the native rapidsnark prover, one Groth16 proof takes 1.45 s. Transparent systems remove the setup altogether [24], but published implementations produce proofs of tens to hundreds of kilobytes with correspondingly slower verification. For this circuit and these implementations, Groth16 with a setup per shape is therefore preferable when the number of shapes is limited and proofs must be fast. A universal setup becomes preferable when shapes change too often to organize a contribution for each.

### 6.9. Comparison with Existing Approaches

Table 7 compares ZK-AdapterMark with representative proof-based systems and their costs. Table A6 in Appendix E lists what each system, proof-based or not, reveals to its verifier and which transformations its authors evaluated. The figures for other systems are taken from their papers. They were obtained on different models, inputs, and hardware; so, the tables support comparisons of order of magnitude and of the kind of statement proved. They do not support exact ratios.

Three observations follow. First, zkLLM and VeriLoRA prove a computation over a whole model. They need minutes of graphics processing unit (GPU) time per proof, their verification takes seconds, and their proofs are about a hundred kilobytes. The Groth16 systems, namely ZKROWNN, the Groth16 variant of PVMark, and ours, produce proofs below one kilobyte that verify in milliseconds, but each circuit needs its own setup. On a central processing unit (CPU) server, one of our layers is proved in 10.4 s into a proof of 706 bytes and verified in 3.8 ms. The six-layer adapter decision takes 58.7 s to prove and 43 ms to verify. Second, among the systems in Table A6, none of the implemented proof-based ones keeps both a secret detection key and the model weights from the verifier, while the test reads the weights. ZKROWNN hides the key but takes the weights as a public input, and the other proof-based systems either have no secret key or test outputs instead of weights. Third, the watermarking and fingerprinting schemes designed for LoRA evaluate robustness in the clear. Two of them, SEAL and Chain & Hash, hand their secret to the verifier during a dispute; so, a copier who observes one dispute learns what to remove.

The experiments thus establish three results. Evaluating the probes on the factors keeps the circuit small even with the commitments included, and the hub’s verification stays fast although proving is still heavy. The fingerprint survives the edits a copier would normally make, and no pair of independently trained adapters is accepted, although single layers of similar adapters reach the threshold. Among the ordinary edits, a merge that leaves the registered update only half the weight marks the boundary, and a deployment should decide deliberately whether that is acceptable. Stronger edits, such as two-bit quantization, and the strategies of Section 6.6 that aim at the fingerprint remove it.

## 7. Related Work

The problem draws on four bodies of work: fingerprints and watermarks for adapters, time-ordered provenance records, privacy-preserving watermark detection, and proofs over low-rank updates. Systems in these areas protect different objects and therefore make different public statements. We compare them by what the verifier receives, what stays hidden, and what an acceptance means, rather than grouping them under a single notion of ownership.

### 7.1. Adapter Fingerprints and Provenance Disputes

Model watermarking encodes a recognizable signal in parameters or behavior [3,4,5,6]. In an ownership dispute, however, persistence and fidelity are only part of the problem; ambiguity and fabricated claims must also be addressed [29,30]. Recent methods focus on parameter-efficient modules and test whether their marks survive additional tuning, merging, or composition with other adapters [31,32,33,34,35,36,37]. All of them verify the mark in the clear, either by reading the adapter or by querying its outputs. We share the aim of a persistent signal. The signed records and zero-knowledge relation deal with the separate question of how that signal is tied to a later file during a dispute.

Chain & Hash most directly shares the concern with time ordering. It registers prompts and expected outputs, compares behavior by a threshold, and orders competing claims, including claims for LoRA adapters [7]. Its verifier queries the model’s behavior. Ours begins with a standalone adapter file in a custodian’s possession and evaluates a hidden test on its weights against a commitment to that file. The two approaches therefore assume different evidence is available at verification time.

Other work strengthens the evidence available for false-claim and lineage questions. FIT-Print derives targeted signatures from black-box fingerprints to separate a protected model from independently trained models [38]. Shang et al. attest fine-tuning lineage from how the knowledge and the parameters of a model evolve together [39]. In our setting, neither a training trace nor the original service is assumed to be available. The observation is a LoRA file obtained independently after the policy was registered.

### 7.2. Private and Publicly Verifiable Detection

Using zero knowledge to hide a watermark detector predates current neural models [19,29]. ZKROWNN develops this idea for neural-network ownership claims [25], ZK-WAGON couples a generative-model watermark with succinct proofs [40], and TIM proves correct extraction of a blind image watermark while keeping the seed and embedding positions private [27]. These systems demonstrate that the public outcome of a detector need not expose its witness, which is the privacy requirement adopted here. None of them targets LoRA adapters, and ZKROWNN materializes dense layers inside the circuit, which is the cost our factor-aware evaluation avoids.

HuRef commits to private language-model parameters and proves the construction needed for a private comparison with an open model [8]. PVMark gives public verification of a secret-key language-model watermark detector [9]. NoisePrints proves a relation between a hidden generation seed and generated media [28]. Xu and Sheng fingerprint a language model by its refusal vector and propose, without implementing it, a zero-knowledge proof that the public hash of this fingerprint was computed from the private parameters [41]. Wilding et al. detect whether a deployed model was modified, for example by LoRA fine-tuning. They search for input probes that amplify the logit drift and prove with Groth16 that the probe outputs match a committed baseline [42]. Their witnesses concern full models, text, or media. ZK-AdapterMark narrows the object to LoRA factors opening a custodian’s commitment, and it evaluates those factors against a threshold fixed in an earlier record.

### 7.3. Proofs over LoRA and Model Updates

The two-factor form of LoRA is exploited well beyond verification, for instance by initialization schemes that calibrate output moments while retaining the factorized parameterization [43]; here the same structure is used to keep a proof small. General zero-knowledge machine learning (ZKML) systems have considered inference, accuracy claims, and large-model computation [26,44]. Proof-of-learning instead verifies evidence from a training history [45]. Systems written specifically for LoRA prove low-rank operations, compatibility, or correct fine-tuning [10,11]. Fine-Tuning Integrity checks structural drift conditions on an update, including low-rank, norm, and sparsity requirements [12].

Our circuit likewise benefits from low rank, although it proves neither a training run nor compliance with an update norm. Its predicate is agreement with hidden directional responses, and the public inputs place this predicate between an earlier registration and a later custodian record so that the low-rank arithmetic yields evidence about one particular candidate file.

## 8. Conclusions

We presented ZK-AdapterMark, a protocol that lets a model hub settle a provenance dispute over a LoRA adapter without learning the fingerprint key or the adapter weights. A registry record fixed before release, a custody record fixed at dispute time, and a zero-knowledge proof of the fingerprint match together bind the verdict to one authenticated file and one prior policy. The circuit enforces the complete per-layer relation, including the registration mode, the context binding, and the Boolean and range checks. The order of the records rests on the registry, and the link between the file and the committed factors rests on the custodian. Evaluating the fingerprint on the two low-rank factors rather than on the dense update keeps the proof circuit more than an order of magnitude smaller than a direct construction; so, proving is a server task of seconds and verification takes milliseconds. Under the fair-bit model, the acceptance threshold bounds the false-accept probability through relative entropy, and for candidates that agree more often than chance, it corresponds to a minimum information per probe.

The experiments confirm that the fingerprint survives the edits a copier is likely to make and that no pair of independently trained adapters is accepted, while an equal-weight merge marks the boundary of the current policy. The evaluation covers persistence on 148 real adapters, separation on 904 adapters trained independently from one base model, and seven adaptive strategies. Robustness varies between adapters, and the variation is explained mainly by how far a transformation turns the update. Section 2.1 delimits the reuse that the evidence addresses. Appendix B states these limitations and the operational requirements that follow from the analysis.

## Figures and Tables

**Table 1:** Components of the per-layer relation and their cost in the implemented circuit at width 768, rank 8, and m=128. Every component is enforced inside the circuit except the checks listed in the last row, which the verifier performs on public values.

Relation Component	Where Enforced	Constraints
Factor-based probe evaluation and sign comparison	Circuit	1,590,784
Probe derivation from $k_{\mathrm{ctx}}$ (Poseidon seeds, canonical bits)	Circuit	1,481,221
Factor commitment $C_{F}$ with μ, ctx, and $\rho_{F}$	Circuit	1,717,441
Test commitment $C_{\mathrm{wm}}$ with ctx and $\rho_{\mathrm{wm}}$	Circuit	1209
Boolean checks on μ and $b_{1},\ldots ,b_{m}$	Circuit	129
Signed 24-bit range checks on all factor entries	Circuit	319,488
Threshold c≥τ+μ(m−τ)	Circuit	16
Total per layer		5,110,288
Signatures, context equality, policy range, and the *k*-of-*L* layer rule	Verifier	—

An em dash marks checks that the verifier performs outside the circuit, which add no constraints.

**Table 2:** Constraint Budget at Deployment Widths (r=8, m=128).

Circuit Content	Width 768	Width 2048
Directional-probe kernel (compiled)	1,590,798	4,212,238
Complete per-layer circuit (compiled)	5,110,288	13,050,934
added by the evidence checks	3.21×	3.10×
Analytic dense reconstruction	80,216,064	570,425,344
reduction over dense	15.7×	43.7×

The host machine does not affect a compiled constraint count. The complete circuit is the one of Section 4.3, with its Boolean and range checks, compiled with circom 2.2.3 at --O1.

**Table 3:** Proof artifacts and cost of the complete per-layer circuit, r=8, m=128, dispute mode, 16 threads. Setup and contribution were measured once, proving times are medians of five proofs, and verification times medians of 30 runs. Memory is the peak resident set.

Width	Constraints	Setup	Contribution	Proof	Native Verify	Resident Verify	Proof File	Proving Key
256	1,932,334	145 s, 6.1 GiB	75 s	3.28 s, 1.2 GiB	3.8 ms	6.2 ms	708 B	0.87 GB
512	3,518,702	263 s, 10.7 GiB	140 s	6.07 s, 2.3 GiB	3.8 ms	6.6 ms	706 B	1.61 GB
768	5,110,288	443 s, 15.9 GiB	245 s	10.4 s, 3.8 GiB	3.8 ms	6.6 ms	706 B	2.49 GB
2048	13,050,934	1435 s, 36.6 GiB	543 s	23.5 s, 8.6 GiB	3.8 ms	6.8 ms	704 B	6.05 GB
768, data-aware	4,831,800	406 s, 15.5 GiB	233 s	9.60 s, 3.5 GiB	3.8 ms	6.3 ms	707 B	2.38 GB

**Table 4:** Registration and dispute of the six-layer DistilBERT adapter, by role, width 768, r=8, m=128.

Role	Step	Time
Claimant	Inputs and witnesses of six layers	20.0 s
	Six registration proofs	58.7 s
Registry	Check of six registration proofs	0.24 s
Custodian	Digest of the 2.5 MB file	8.8 ms
	Decoding of the factors	24 ms
	Encoding and commitments	2.01 s
	Signature	2.7 ms
Prover	Inputs and witnesses of six layers	20.2 s
	Six proofs, sequential	58.7 s
	Six proofs, two concurrent jobs	56.0 s
	Six proofs, six concurrent jobs	55.6 s
Verifier	Check of the records	2.0 ms
	Six proofs, resident snarkjs	43 ms
	Decision	accept, 6 of 6

**Table 5:** DistilBERT Fingerprint Agreement After Transformation.

Candidate Change	Agreement	Range
8-bit quantization	127.5±0.6	126–128
4-bit quantization	119.0±3.7	112–124
3-bit quantization	108.5±4.5	97–115
2-bit quantization	77.3±5.7	67–88
Remove 1 of 4 ranks	122.3±3.8	113–128
Remove 2 of 4 ranks	116.5±7.3	101–126
Keep 1 of 4 ranks	102.6±13.4	79–125
Noise, σ=0.0046	117.7±3.7	110–123
Noise, σ=0.01	104.2±3.1	98–110
Noise, σ=0.015	98.0±5.1	89–106
Noise, σ=0.022	86.1±5.9	71–97
Merge, α=0.9	126.2±3.1	115–128
Merge, α=0.7	114.6±5.7	107–127
Merge, α=0.55	101.5±3.8	93–108
Merge, α=0.5	95.6±4.4	87–102
Merge, α=0.45	90.2±6.4	78–99
Unrelated random factors	65.0±5.4	55–73

Mean, standard deviation, and range of the agreement count out of 128 over 18 layer-key observations. Noise levels are the nearest points of the log-spaced sweep; α is the weight of the registered update in the merge.

**Table 6:** Separation from independently trained adapters, isotropic probes, m=128, τ=96, accepted when at least five of six layers pass. The column “most layers” gives the largest number of layers that any pair passes under one key. Lots-of-LoRAs and LoRA Land were compared under three keys and the other collections under five, once per unordered pair and key. SD: standard deviation of the layer-level count.

Collection	Adapters	Layer Comparisons	Mean	SD	Max	Most Layers	Adapter Accepts
Lots-of-LoRAs, q_proj	904	7,346,808	64.2	5.70	101	1	0 of 1,224,468
Lots-of-LoRAs, v_proj	904	7,346,808	64.4	5.79	110	3	0 of 1,224,468
LoRA Land, q_proj	26	5850	64.7	5.86	85	0	0 of 975
DistilBERT, IMDB seeds	10	1350	65.3	5.64	86	0	0 of 225
RoBERTa, five GLUE tasks	9	1080	64.9	5.94	85	0	0 of 180
Qwen2.5-0.5B, four tasks	6	450	64.3	5.77	82	0	0 of 75
GPT-2, seeds and corpora	5	300	75.5	14.97	112	4	0 of 50
Matched-statistics controls	—	540,000	64.0	5.65	91	—	—
Bin(128,1/2)	—	—	64	5.66	—	—	—

**Table 7:** Proof-based systems: object of the proof and reported cost. n.r.: not reported in the cited paper. n.a.: not applicable; KZG: Kate–Zaverucha–Goldberg polynomial commitment; FTI: Fine-Tuning Integrity. Our rows use the complete circuit of Section 4.3; the dense baseline is the circuit that rebuilds BA before probing. For six layers, the proof size is the total of the six proof files, and verification uses a resident snarkjs verifier.

System	Object of the Proof	Proof System (Setup)	Circuit Size	Proof Size	Proving Time	Verification	Hardware
ZKROWNN [25]	DeepSigns watermark extraction, MNIST MLP	Groth16 (per circuit)	$2.09\times 10^{6}$ constraints	127 B	45.1 s	29.4 ms	Ryzen 3990X, 128 GB
zkLLM [26]	Inference of OPT-125M to LLaMA-2-13B	Sumcheck, Hyrax (none)	n.r.	141–188 kB	73.9–803 s, plus 11.8–1270 s commitment	0.34–3.95 s	A100 GPU, 12 CPU cores
ZKLoRA [10]	LoRA module outputs in shared inference	Not stated	n.r.	n.r.	n.r. (no units given)	1–2 s per module	n.r.
VeriLoRA [11]	One LoRA fine-tuning step, 3B–13B models	Sumcheck, lookups, Hyrax (none)	n.r.	n.r.	122–249 s, plus 156–554 s commitment	1.87–3.73 s	A100 GPU, 192 CPU cores
PVMark [9]	Text watermark detection, 200 tokens	Groth16, PlonK, halo2, and Nova	n.r.	0.8, 2.3, 14, and 10.2–10.5 KB	about 24 s (Nova, setup and proof)	below 2 s	Xeon Gold, RTX 3090
TIM [27]	Image watermark extraction, 4K	Nova and Spartan (none)	$9.7\times 10^{4}$ per step	n.r.	5.61 min	about 0.75 s	i9-14900K, 48 GB
NoisePrints [28]	Seed–image correlation above a threshold	Mirage	n.r.	at most 356 B	0.77 s per chunk, plus 0.92 s	0.42 s plus 0.12 s	Threadripper 5995WX
FTI [12]	Update within a rank 8 class, GPT-2	Custom protocol, simulated KZG	n.a.	1.7 MB	6.2 min, one core, Python prototype	10.5 ms	Apple M-series laptop
Ours, one layer	Keyed fingerprint match of LoRA factors, width 768, r=8	Groth16 (per shape)	$5.11\times 10^{6}$ constraints	706 B	10.4 s	3.8 ms	16 vCPU AMD EPYC, 64 GB
Ours, six layers	Same, adapter-level decision	Groth16 (per shape)	$6\times 5.11\times 10^{6}$	4248 B	58.7 s, sequential	43 ms	16 vCPU AMD EPYC, 64 GB
Dense baseline	Same statement, BA rebuilt in the circuit	Groth16 (per shape)	$8.0\times 10^{7}$ (analytic)	—	not feasible	—	—

**Figure A2:**
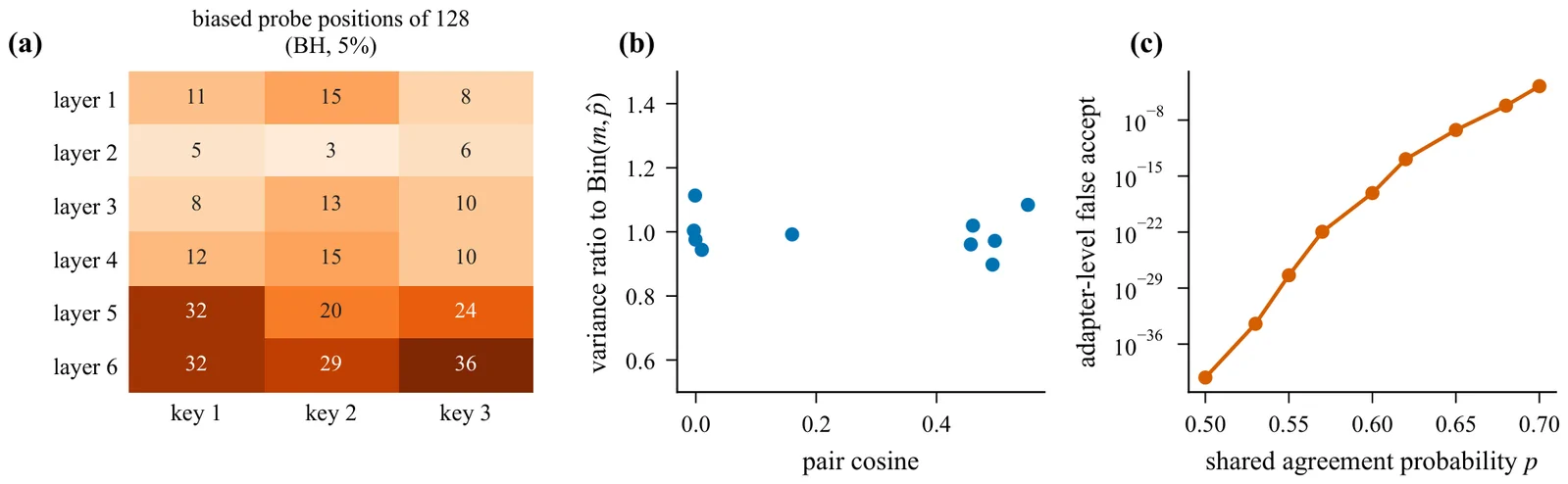
The fair-bit model tested on 904 independently trained Lots-of-LoRAs adapters. (**a**) Number of the 128 probe positions whose fraction of ones differs significantly from 1/2 after Benjamini–Hochberg control, per layer and key. (**b**) Variance in the count over 1000 keys relative to that of $\mathrm{Bin}(m,\hat{p})$ for ten pairs, against the cosine of the pair; the dispersion tests give *p*-values between 0.01 and 0.92. (**c**) Adapter-level false-accept probability of the five-of-six rule when every layer has the same agreement probability *p*; the plug-in estimate from the measured distribution of *p* is $4.8\times 10^{-15}$, and the angle law gives $9.3\times 10^{-15}$.

## Data Availability

Data are contained within this article.

## Appendix A. How the Measurements Were Obtained

This appendix describes how the numbers of Section 6 were obtained so that a reader can judge them and repeat them. Table A1 shows which circuit or experiment each kind of number comes from.

### Appendix A.1. Circuit, Server, and Timing

Every timing, memory figure, proof size, and key size in this paper comes from one version of the complete circuit of Section 4.3. The circuit was compiled with circom 2.2.3 at --O1 together with its C++ witness generator. Proofs were generated with the native prover rapidsnark using 16 threads, and setup, contribution, and verification used snarkjs 0.7.6. All of these ran on one Alibaba Cloud server with 16 vCPUs on 8 AMD EPYC cores and 64 GiB of memory under Ubuntu 22.04, and no other job ran during the measurements. Proving times are medians of five proofs, resident and native verification times medians of 30 runs, and fresh-process verification times medians of six runs; setup and contribution were measured once. Peak memory is the maximum resident set reported by GNU time. The compile-only sweeps and the plaintext fingerprint experiments ran on servers of the same kind. They produce no proofs; so, the host does not affect their results. Training and the attack experiments used one NVIDIA A10 GPU.

One change to the circuit came after these measurements. The probe derivation now uses the low 252 bits of each Poseidon output instead of 253, because bit 252 of a uniform field element equals one with probability 0.339, like bit 253. The change alters which bits feed the directions and leaves every constraint count unchanged, since ⌈d/252⌉=⌈d/253⌉ for every timed width. The changed circuit passed all positive and negative tests, and its compiled counts at widths 768 and 2048 equal those of Table 3. A rerun at width 768 took 470 s for the initial key and 10.3 s per proof, against 443 s and 10.4 s in the same table. The six-layer run of Table 4 again accepted all six layers, with six sequential proofs in 58.6 s against 58.7 s.

**Table A1:** Sources of the Reported Measurements.

Artifact	Measurements Reported from It
Direct comparison	Constraint counts for two circuits computing the same directional scores, with and without dense reconstruction, at d=128, r=4, m=16
Probe kernel	Constraint counts for the directional-test kernel alone, at d∈{768,2048}, r=8, m=128
Complete per-layer circuit	Constraint counts, setup, contribution, proving, verification, proof files, and keys at widths 256, 512, 768, and 2048; registration mode and the data-aware variant at 768; registration and dispute of the six-layer DistilBERT adapter
Universal-setup comparison	Groth16, PLONK, and FFLONK at width 64 under the snarkjs prover
Plaintext sweeps	Agreement along the noise, quantization, rank, and merge sweeps; layer and adapter decisions; 540,000 unrelated controls; mutual-information and tail curves; both adapters
Compile-only sweep	Constraint counts of the probe kernel, the dense baseline, the building blocks, and the complete circuit at the widths, ranks, and fingerprint lengths in Figure 3; no proofs generated

### Appendix A.2. Constraint Counts

The constraint sweeps in Figure 3 compile the complete circuit of Section 4.3, whose template takes the layer shape, the rank, and the fingerprint length as parameters. The probe kernel and the dense circuit in the figure come from an earlier sweep with the same kernel template.

To see where the constraints go, we also compiled the seven building blocks of the complete circuit as separate circuits at every width of Figure 3d. They are the probe kernel, the probe derivation from $k_{\mathrm{ctx}}$, the factor commitment, the test commitment, the Boolean checks, the 24-bit range checks, and the threshold. At every width their counts add up to the compiled total of the complete circuit. The kernel inside the complete circuit has $m\left(r(d_{\mathrm{out}}+d_{\mathrm{in}})+140\right)$ constraints. Kernel counts from earlier compilations exceed this value by 14 to 38 constraints, less than 0.003 percent, which affects no ratio in the text.

Section 6.8 also needs estimates for shapes that were not compiled. For these we fitted $c_{0}+k_{1}mrw+k_{2}mw+k_{3}rw$ by least squares to 18 compiled counts, with $w=d_{\mathrm{out}}+d_{\mathrm{in}}$. The points comprise eight widths, three real layer shapes of GPT-2 and Qwen2.5, and seven rank and fingerprint-length variants. On these points the fit has a mean relative error of 1.4 percent and a largest error of 9.9 percent, at width 128. Among the real shapes, the GPT-2 c_attn projection, 2304×768 at rank 8, has 9,875,986 constraints. Two real shapes compiled after the fit, the query and the key and value projections of Llama-3.2-1B at rank 16, have 22,668,028 and 14,296,294 constraints, and the fit predicts both within 0.2 percent.

### Appendix A.3. Fingerprint Experiments

For persistence, we evaluated three independent keys on each of six layers from one task-trained DistilBERT adapter, giving 18 observations per setting, and three keys on each of 12 synthetic rectangular layers with $d_{\mathrm{out}}=2304$, $d_{\mathrm{in}}=768$, and rank 8, giving 36. The DistilBERT directions are the Poseidon-derived vectors of the timed circuit, which took the low 253 bits of each output. The GPT-2-shaped directions come from the HMAC-SHA-256 Rademacher generator of the plaintext reference implementation, since the Poseidon directions were exported only for width 768. Each layer-key pair contributes 10,000 unrelated controls matched to its factor statistics, 540,000 in all. The sweeps involve no proof generation; so, the host does not affect them. Quantization and rank reduction are deterministic; so, the sweeps reproduce the counts of these settings exactly. Noise and merging draw random partners, and redrawing them moves the mean count by at most about two agreements. The real-adapter experiments of Section 6.3, Section 6.4, Section 6.5, Section 6.6 and Section 6.7 use the HMAC-SHA-256 Rademacher generator of the plaintext reference. Under the pseudorandomness assumptions on both generators, its directions have the same distribution as those of the Poseidon generator. The experiments therefore model the probes of the circuit statistically and do not reproduce them bit for bit. Across the 2220 factor tensors of 99 of these adapters, the largest magnitude is at most 85 times the root-mean-square (RMS) value; so, the RMS value spans at least 24,000 encoding steps. In all 2664 checks, one per adapter, key, and layer, the encoded adapter kept 128 of 128 agreements with the floating-point responses.

## Appendix B. Limitations and Operational Considerations

This appendix gathers the limits of the evidence and the requirements that a deployment has to meet, from the scope of the experiments to the meaning of an accepted verdict.

### Appendix B.1. Scope of the Empirical Evidence

The persistence and separation measurements cover 148 real adapters in ten model families and 904 adapters trained independently from one base model. They show that the agreement is governed by the update angle. Whether a threshold suits a particular deployment should still be calibrated on adapters of that deployment.

Section 6.6 measured agreement and task utility together after merging. When the RoBERTa adapter for SST-2 is merged linearly with adapters of other tasks, it is accepted as long as its weight is at least 0.4. At that weight the accuracy is 0.908, against 0.932 for the original. At weight 0.3 the accuracy is 0.894 and the adapter is rejected. For this adapter the boundary therefore lies between weights 0.3 and 0.4. A deployment should evaluate agreement and task utility together on the same transformed candidates.

### Appendix B.2. Retraining Adversaries

Section 2.1 places adversaries that set out to remove the fingerprint outside the scope of this paper. This boundary is not specific to our scheme. Retraining produces a new update whose angle to the registered one can exceed 45°, and our fingerprint then no longer separates the copy from an independently trained adapter. The systematization of Lukas et al. found that every model watermark it evaluated could be removed by at least one attack at a small cost in utility [13]. Watermarks designed to survive model extraction, such as entangled watermarks [46], raise the cost of removal but have to modify the model during training. Methods that do not read the weights, such as dataset inference [47], test whether a model has learned the owner’s private training data; so, they also apply to distilled models. They need query access to the model and the disclosure of some private samples, and proving them in zero knowledge requires proving inference over the model, which zkLLM reports to take minutes on a GPU [26].

Within this boundary the scheme remains useful in three ways. First, the reuse seen most often, direct redistribution, quantization, pruning, and merging, is recognized reliably in Section 6.3, Section 6.4, Section 6.5 and Section 6.6, and the verifier sees neither the weights nor the key. Second, zero knowledge keeps the key secret. With public probes a reuser could target the measured directions. With a secret key and the balanced form of Section 4.4, the adversary has to change the update itself, which in the attacks of Section 6.6 required task data. Third, the proved relation does not depend on the particular test. It proves that a previously registered test passes on the file the custodian recorded; so, a more robust test can replace the current one within the same protocol. Against retraining adversaries, weight evidence can be combined with data-based evidence, which we leave to future work.

### Appendix B.3. Scope of the Circuit

The circuit enforces the complete per-layer relation of Equation (A4), including the registration mode, the context binding, the Boolean constraints, and the range checks described in Section 4.3. The signatures, the context equality between the two records, the admissibility of the policy, and the adapter-level rule are checked by the verifier outside the circuit. The order of the records rests on the registry log, and the correspondence between the file and the candidate commitment rests on the custodian, as Section 2.4 describes. A security audit must therefore cover the full acceptance procedure of Equation (A2), including these checks outside the circuit.

### Appendix B.4. Repeated Queries and Secret Access

The verdict leaks a small amount of information. An attacker who can submit many adjusted candidates can probe the acceptance boundary from the accept-or-reject outcomes. A service should therefore authenticate requests, retain candidate digests, and limit repeated trials under one policy. A fresh context changes the probes and the commitments; so, a claimant who wants one for later examinations must register several contexts in advance. A context registered later needs a new registration, which does not inherit the time of the earlier one. Zero knowledge protects the witness in each proof, but it does not conceal patterns that become visible across many decisions.

Confidential mode hides the weights from the verifier, not from the prover. The authorized proving environment receives both the fingerprint key and the candidate factors in order to construct the witness. Avoiding their exposure to any single proving party would require joint proving, delegated computation, or trusted hardware.

### Appendix B.5. Meaning of an Accepted Verdict

An accepted verdict states only that the file the custodian named passes a policy registered earlier. It does not reveal how the file was obtained or who originally created it. Questions of ownership, authorship, licence obligations, or lawful distribution require evidence that is not represented in the proof relation, and a deployment should report the verdict in those terms rather than as ownership verified.

## Appendix C. Data-Aware Probes

Section 6.6 found that the low-variance perturbation removes the fingerprint because the isotropic probes also cover input directions that the model hardly uses. This appendix describes optional probes that avoid those directions and reports how they behave.

Data-aware probes change only the input directions. Let *S* be the second-moment matrix of a layer input on a public corpus, computed with the base model. Let $E_{k}$ hold the leading *k* eigenvectors of *S* and $\Lambda_{k}$ the corresponding eigenvalues, where *k* is the smallest number that keeps 99 percent of the trace. The *i*-th input direction is $v_{i}=E_{k}\Lambda_{k}^{1/2}w_{i}$, where $w_{i}\in {\left\{-1,+1\right\}}^{k}$ is derived from the layer key; the isotropic probes are the special case $E_{k}=I$, $\Lambda_{k}=I$. $E_{k}$ and $\Lambda_{k}$ depend only on the base model and the public corpus; so, they can be fixed in public, and their digest enters the context. In the circuit, $P=E_{k}\Lambda_{k}^{1/2}$ is a constant matrix; so, the product AP adds only linear constraints, and the probe inner products need mrk multiplications instead of $mrd_{\mathrm{in}}$. The entries of *P* are encoded with the power-of-two rule of Section 4.4; so, they are integers of absolute value below $2^{22}$. With factor entries of absolute value at most $2^{23}$, every score then has absolute value below $r\;d_{\mathrm{out}}\;d_{\mathrm{in}}\;k\;2^{68}$. This is below $2^{100}$ at width 768, rank 8, and k=661; so, the 128-bit sign extraction stays exact. Since *P* is compiled into the circuit, each base model and corpus gives its own circuit and setup, and the shape counts of Section 6.8 cover only the isotropic probes. Proposition 3 applies with XP and YP in place of *X* and *Y*. For the first attention layer of DistilBERT, computed on the IMDB training texts, k=661 with $d_{\mathrm{in}}=768$. At width 768 and rank 8 the circuit then has 4,831,800 constraints, 5.4 percent fewer than the isotropic circuit. This variant passes all positive and negative tests of the complete circuit of Section 4.3. Figure A1 compares the two kinds of probes under the low-variance perturbation and the benign edits.

## Appendix D. Protocol Details and Proofs

This appendix collects the formal parts of Section 2, Section 3, Section 4 and Section 5: the protocol syntax, the analysis of compromised parties, the relation proved for each layer, the encoding of the factors, and the proofs.

### Appendix D.1. Protocol Syntax

The workflow of Section 2 can be stated compactly as five algorithms:

(A1) Π=(Setup,Register,Fix,Prove,Verify).

The version identifier *v* fixes all details that affect a circuit statement: dimensions, fingerprint length, integer encoding, hash construction, and proof keys. We write $F_{0}={\left\{\left(B_{0,\ell },A_{0,\ell }\right)\right\}}_{\ell }$ for the original factors included in a policy, k for their keys, and ctx for the corresponding contexts. The symbols pol and ser denote the adapter policy and canonical serialization rule. Registry and custodian signing keys are $sk_{R}$ and $sk_{C}$, with public keys $pk_{R}$ and $pk_{C}$. In the syntax below, values following a semicolon remain private to the claimant during the interactive registration protocol.

- $\mathrm{Setup}\left(1^{\lambda },v\right)\;\to \;\left(pp_{v},pk_{v},vk_{v}\right)$ publishes the parameters and selects the proving and verification keys belonging to version *v*.
- ${\mathrm{Register}}^{sk_{R}}\left(pp_{v},pk_{v},\mathrm{ctx},\mathrm{pol};F_{0},k\right)\;\to \;\mathrm{rec}$ returns the prior record. From its private inputs, the claimant constructs commitments and the consistency proofs $\left\{\pi_{\mathrm{reg},\ell }\right\}$. The registry checks their public statements, then signs the contexts, commitments, thresholds, versions, and policy.
- ${\mathrm{Fix}}^{sk_{C}}\left(\mathrm{file},\mathrm{ctx},\mathrm{ser}\right)\;\to \;\mathrm{cand}$ returns $\mathrm{cand}=(D^{*},\mathrm{ser},{\left\{\left({\mathrm{ctx}}_{\ell },v_{\ell },C_{\mathrm{cand},\ell }\right)\right\}}_{\ell },\sigma_{C})$ for the file processed by the custodian.
- $\mathrm{Prove}\left(pk_{v},x_{\ell },w_{\ell }\right)\;\to \;\pi_{\ell }$ runs inside the authorized environment and produces the private-match proof for layer *ℓ*.
- $\mathrm{Verify}\left(pk_{R},pk_{C},\left\{vk_{v}\right\},\mathrm{rec},\mathrm{cand},\left\{\pi_{\ell }\right\}\right)\;\to \;\left\{0,1\right\}$ checks the signatures, rebuilds the proof statements, verifies the supplied proofs, and evaluates the signed policy.

Acceptance combines the five operations above. Once rec and cand have been authenticated, the verifier reconstructs $x_{\ell }=\left(0,{\mathrm{ctx}}_{\ell },C_{\mathrm{cand},\ell },C_{\mathrm{wm},\ell },\tau_{\ell }\right)$ and computes:

(A2) $$\begin{array}{cc} a_{\ell } & =\mathrm{ZKVerify}(vk_{v_{\ell }},x_{\ell },\pi_{\ell }),\\ \mathrm{Accept} & =\mathrm{RegValid}(\mathrm{rec})\wedge \mathrm{CandValid}(\mathrm{cand})\\ \; & \;\wedge \mathrm{ContextEq}(\mathrm{rec},\mathrm{cand})\\ \; & \;\wedge \mathrm{LayerPolicy}(\mathrm{pol},\left\{\left(\ell ,a_{\ell }\right)\right\}). \end{array}$$

Here, RegValid covers the registry signature and the registration consistency proofs, whereas CandValid covers the custodian signature and canonicalization metadata. ContextEq rejects a bundle if the records disagree on the model, layer, dimensions, encoding, or circuit version. LayerPolicy also checks that a layer is not counted twice and that the recorded minimum number of layers has passed. It also checks that the signed policy is admissible, with $1\leq \tau_{\ell }\leq m$, 1≤k≤L, and distinct layers, since a threshold or a layer count of zero would let any candidate pass. A layer counts as passing only when its proof verifies. A missing or invalid proof leaves the layer unestablished, which is weaker than evidence that the layer fails the test. Section 3 and Appendix D.3 define the relation from which each $a_{\ell }$ is obtained.

### Appendix D.2. Analysis of Compromised Parties

A verdict depends on several parties, and each of them can deviate from the protocol. This subsection states which guarantees remain when they do. We name five guarantees, G1 to G5. G1 is prior registration: the registered policy existed before the disputed file was fixed. G2 is file binding: the candidate commitment was computed from the file named in the candidate record. G3 is match soundness: an accepted proof implies that the committed candidate passes the committed test. G4 is test privacy: the verifier learns nothing about the key or the reference responses beyond the public records and the outcome of each layer. G5 is weight privacy: in the confidential mode, the verifier learns nothing about the candidate factors beyond their commitments and the layer outcomes. G3 follows from cryptographic assumptions. G4 and G5 follow from them as well when the commitments are hiding and the prover follows the protocol. G1 rests on the registry, and G2 rests on the custodian. G1 orders the registration before the custodian’s record. Whether the registration also precedes the first release of the disputed file is a separate question, which the hub can answer from its own upload records.

A dishonest custodian can misrepresent the processed file. It can sign a commitment to factors that do not come from the named file, for example the claimant’s original factors, and an unrelated file would then be accepted. The damage depends on the mode. In the public mode the file can be downloaded, the decoding procedure is fixed by the circuit version, and the blinding value of the candidate commitment is published with the record. Any verifier can recompute the commitment from the file and compare it with the signed value; so, a false record is detected by the first verifier who checks it. The custodian is therefore not needed in this mode, because the verifier can build the candidate record itself. In the confidential mode the verifier cannot recompute the commitment, and G2 rests on the custodian. Two measures reduce this dependence. The party that uploaded the disputed file holds the same bytes, and if it receives the blinding value it can recompute the commitment and contest a substitution. The candidate record can also require signatures from *k* of *n* independent custodians that decode the file separately, or an attestation that the decoding ran inside a trusted execution environment [48]. If all custodians collude, G2 does not hold in the confidential mode.

A dishonest registry can assign a record an earlier position than the one at which it arrived so that a policy created after the disputed file appeared seems to predate it. This breaks G1. An append-only transparency log makes such backdating detectable. The registry publishes a signed digest of the whole log at regular intervals, either to independent witnesses or to a time-stamping authority [49]. A record inserted into an earlier part of the log then changes a digest that others already hold. A consistency proof between two published digests reveals the change [50,51]. A registry can also refuse or delay a registration. This harms availability but not correctness, and signed receipts let the claimant prove the refusal. The registry never receives the key, the reference responses, or the factors; so, it cannot break G4 or G5.

The prover and the setup complete the list. A dishonest prover cannot make a false statement verify, because G3 follows from knowledge soundness and does not depend on the prover. In the confidential mode, however, the prover receives the key and the candidate factors; so, G4 and G5 hold toward the verifier but not toward the prover, and a prover can disclose what it holds. Groth16 soundness assumes that the secret randomness of the circuit-specific setup was destroyed. An adversary who knows it can produce accepting proofs for false statements, and G3 then fails for every circuit shape built from that setup. A phase 2 ceremony with several independent contributors reduces the requirement to one honest contributor per shape [21]. Zero knowledge can survive a dishonest setup as well. Under the knowledge assumptions of Fuchsbauer [52], it holds when the prover checks the proving key with the verification algorithm of that work before it proves.

Table A2 lists these cases together with the coalitions. A claimant who colludes with the custodian can obtain a false accept in the confidential mode. A claimant who colludes with the registry can backdate a policy unless the log is witnessed. A custodian who colludes with the uploader of the disputed file can cause a false reject by committing to an unrelated file. If the claimant, the registry, and the custodian all collude, they can produce an accepting bundle for any file. The proof then remains sound, but it concerns records that no longer describe what happened, and only the privacy guarantees toward the verifier remain. A deployment should therefore select registries and custodians whose interests are independent of the claimant’s.

**Table A2:** Guarantees that remain when parties deviate from the protocol. G1: prior registration; G2: file binding; G3: match soundness; G4: test privacy; G5: weight privacy in the confidential mode. Privacy guarantees are stated toward the public verifier.

Deviating Parties	Possible Attack	Guarantees Kept	Detection or Mitigation
Custodian, public mode	Commits to factors of another file	G1, G3–G5; G2 restored by recomputation	Any verifier recomputes the candidate commitment; the custodian can be omitted
Custodian, confidential mode	Commits to factors of another file	G1, G3–G5	Uploader recomputes the commitment; *k*-of-*n* custodians; attested decoding
Registry	Backdates, refuses, or delays a record	G2–G5	Witnessed transparency log; signed receipts
Prover	Refuses to prove; sees the key and the factors in the confidential mode and can disclose them	G1–G3; G4, G5 unless the prover discloses	The claimant proves; proving in an attested environment
Setup contributors	Forge proofs for one circuit shape	G1, G2; G4, G5 if the prover checks the key	Multi-party phase 2 ceremony; key check by the prover
Claimant and custodian	False accept of an unrelated file in the confidential mode	G1, G3–G5	As for the custodian; custodians independent of claimants
Claimant and registry	Backdated policy	G2–G5	As for the registry
Uploader and custodian	False reject through an unrelated file	G1, G3–G5	*k*-of-*n* custodians; attested decoding
Claimant, registry, and custodian	Accepting bundle for any file	G3–G5, without provenance meaning	None within the protocol

### Appendix D.3. Unified Registration and Dispute Relation

Registration and dispute check the same computation and differ only in how many bits must agree; so, one relation can serve both by means of a bit μ∈{0,1}. With μ=1, used at registration, all responses must agree. With μ=0, used for a later candidate, the recorded threshold is enough. The public statement and private witness are:

(A3) $$\begin{array}{cc} x_{\mu } & =(\mu ,\mathrm{ctx},C_{F},C_{\mathrm{wm}},\tau ),\\ w_{\mu } & =(B,A,k,b_{1},\ldots ,b_{m},\rho_{F},\rho_{\mathrm{wm}}). \end{array}$$

Accordingly, $C_{F}$ refers to the original-factor commitment when μ=1 and to the custodian’s candidate commitment when μ=0. The blinding values $\rho_{F}$ and $\rho_{\mathrm{wm}}$ make the commitments hiding; Section 4.3 describes how they are chosen in the two modes. Leaving the fixed domain tags and serialization notation implicit, the condition $R_{\mu }\left(x_{\mu },w_{\mu }\right)=1$ is:

(A4) $$\begin{array}{cc} C_{F} & ={\mathrm{Com}}_{F}\left(\mu ,\mathrm{ctx},B,A;\rho_{F}\right),\\ C_{\mathrm{wm}} & ={\mathrm{Com}}_{\mathrm{wm}}\left(\mathrm{ctx},k,b,m,\tau ;\rho_{\mathrm{wm}}\right),\\ \left(u_{i},v_{i}\right) & =G_{k}\left(\mathrm{ctx},i\right),\;b_{i}^{\prime }=1\left[\left(u_{i}^{T}B\right)\left(Av_{i}\right)\geq 0\right],\\ c & =\sum_{i=1}^{m}1\left[b_{i}=b_{i}^{\prime }\right]\geq \tau +\mu \left(m-\tau \right). \end{array}$$

The lower bound in the last line equals *m* in registration mode and τ in dispute mode. The circuit must also prove that μ and all response variables are Boolean. Range constraints are equally important: without bounds on signed factor encodings and intermediate sums, a valid field equality could correspond to an integer computation that wrapped modulo the field.

Notice that $R_{\mu }$ says nothing about custody or registry order. Those meanings come from the verifier’s checks of signatures, context equality, and the multi-layer policy in Equation (A2). This separation is intentional. Arithmetic secrets, namely the key, the reference responses, and the confidential factors, stay in the witness, whereas the origin and consistency of the two records remain visible to an auditor.

### Appendix D.4. Encoding of Factors and Probe Signs

Before they enter the witness, the factors are brought into a balanced form that depends only on their product. With thin QR decompositions $B=Q_{B}R_{B}$ and $A^{T}=Q_{A}R_{A}$ and a singular value decomposition $R_{B}R_{A}^{T}=U\Sigma V^{T}$, the balanced factors are $Q_{B}U\Sigma^{1/2}$ and $\Sigma^{1/2}V^{T}Q_{A}^{T}$. Their product is still BA. Each singular pair is signed so that the entry of largest magnitude in its column of the first factor is positive. A refactorization $\left(BQ,Q^{-1}A\right)$ of the same update therefore yields the same balanced factors, up to ties among singular values and the floating-point precision fixed by the version. Without this step, a badly conditioned *Q* could push parts of the factors below the resolution of the encoding. The values of each balanced factor tensor are then multiplied by a power of two and rounded to the nearest integer, with halves rounded up. The power is chosen from the largest magnitude in the tensor so that this magnitude lies between $2^{21}$ and $2^{22}$, inside the signed 24-bit range. Scaling a factor by a positive number scales every score by the same positive number. The response signs therefore do not depend on the chosen power, and one rule serves adapters whose factor values differ by orders of magnitude. The rule is deterministic; so, anyone who decodes the same file obtains the same integers. Negative integers are then mapped to field elements according to the signed encoding defined by that version, which represents a negative integer by its sum with the field order.

The margin condition of Section 3.1 keeps small differences in this encoding from changing a sign. Let $\hat{B}$ and $\hat{A}$ be the encoded factors. Suppose that another decoding of the same update gives $\hat{B}+D_{B}$ and $\hat{A}+D_{A}$ with the same powers of two, and that every entry of $D_{B}$ and $D_{A}$ has absolute value at most one. This covers a refactorization of the same update, whose balanced factors differ from the registered ones only by floating-point error. For a probe (u,v) the integer score then changes by at most $\Delta \left(u,v\right)=d_{\mathrm{out}}∥\hat{A}{v∥}_{1}+d_{\mathrm{in}}{∥{\hat{B}}^{T}u∥}_{1}+r\;d_{\mathrm{out}}\;d_{\mathrm{in}}$, since these three numbers bound the three terms of $u^{T}D_{B}\hat{A}v+u^{T}\hat{B}D_{A}v+u^{T}D_{B}D_{A}v$ in this order. If every registered score $s=u^{T}\hat{B}\hat{A}v$ satisfies |s|>Δ(u,v), every such decoding reproduces all registered signs. This sufficient margin is checked on integers before registration and needs no constraint in the circuit.

The probe signs come from Poseidon outputs. Every hash output is decomposed canonically into 254 bits, and its low 252 bits become 252 consecutive signs of a direction; so, a direction of length *d* uses ⌈d/252⌉ outputs. The two highest bits are not used, because each of them equals one with probability 0.339 for a uniform field element. Each of the remaining bits equals one with probability between 0.4959 and 0.5.

### Appendix D.5. Proofs

This subsection proves the statements of Section 4 and Section 5. The lemma on wrap-around comes first, because Proposition 1 assumes its conclusion.

**Lemma** **A1** (No wrap-around)**.** *Let every entry of B and A be an integer of absolute value at most $2^{23}$, and let u and v have entries in {−1,+1}. Then, every partial sum that the circuit forms while computing $s=\left(u^{T}B\right)\left(Av\right)$ has absolute value at most $r\;d_{\mathrm{out}}\;d_{\mathrm{in}}\;2^{46}$. For every shape of Table A4, with ranks up to 2048 and dimensions up to 18,944, this bound is at most $2^{81}$, far below half of the BN254 scalar field order, which exceeds $2^{253}$. The field computation therefore equals the integer computation, and a sign extraction over N bits is exact whenever $2^{N-1}$ exceeds the bound.*

**Proof.** Each entry of $u^{T}B$ is a sum of $d_{\mathrm{out}}$ terms of absolute value at most $2^{23}$, and each entry of Av is a sum of $d_{\mathrm{in}}$ such terms. The score is a sum of *r* products of one entry of each. Every partial sum of these three expressions obeys the same bound as the complete sum.    □

**Proof of Proposition** **1.** The scalar equality is the associativity identity of Equation (5). For the first count, sum the work in two matrix-vector projections and one length-*r* inner product. A secret sign $u_{j}=2t_{j}-1$ with a bit $t_{j}$ turns each term $u_{j}B_{ja}$ into $2t_{j}B_{ja}-B_{ja}$, one product of two witness values, which gives the second count. The third count is the standard cost of multiplying the two dense dimensions through rank *r*.    □

**Proof sketch of Proposition** **2.** Unforgeability assigns the two public records to their stated registry and custodian. Commitment binding rules out replacing an opening after either record has been signed. Knowledge soundness for an accepted layer proof supplies factors, a key, and response values satisfying the two openings, the keyed probe calculation, and the threshold. Zero knowledge hides those extracted values from the verifier. Finally, Equation (A2) requires identical contexts across the records and proofs before evaluating the signed layer rule. The accepted bundle therefore has properties (i)–(iii).    □

**Proof sketch of Proposition** **3.** Given *u*, both scores are sums of $d_{\mathrm{in}}$ independent terms $x_{j}v_{j}$ and $y_{j}v_{j}$ with $Ev_{j}=0$ and $Ev_{j}^{2}=1$. Under the stated condition the multivariate central limit theorem applies, and the pair is approximately normal with covariance entries ${∥x∥}^{2}$, 〈x,y〉, and ${∥y∥}^{2}$. For a centered bivariate normal pair with correlation ρ, both components have the same sign with probability 1−arccos(ρ)/π [53]. The two expectations follow from $E[uu^{T}]=I$. For updates of low rank, $\rho_{u}$ varies with *u*, and Equation (8) is then only an approximation whose error depends on the updates. Section 6.4 measures this error on real adapters.    □

## Appendix E. Additional Results

This appendix gives the tables and figures that support Section 6 but are not needed to follow it.

### Appendix E.1. GPT-2-Shaped Stress Test

Section 6.3 also transformed a synthetic adapter with the rectangular shape of a GPT-2 attention projection. Table A3 gives its agreement counts for the same settings as Table 5.

**Table A3:** GPT-2-Shaped Rank 8 Stress Test.

Candidate Change	Agreement	Range
8-bit quantization	127.7±0.5	126–128
4-bit quantization	124.1±1.6	120–127
3-bit quantization	118.9±2.3	113–123
2-bit quantization	103.2±4.0	95–113
Remove 1 of 8 ranks	115.6±3.0	109–121
Remove 4 of 8 ranks	96.8±4.0	88–105
Keep 1 of 8 ranks	76.5±5.2	59–85
Noise, σ=0.0046	119.0±2.2	115–124
Noise, σ=0.01	109.9±3.9	104–121
Noise, σ=0.015	101.4±4.7	89–109
Noise, σ=0.022	95.0±4.8	83–107
Merge, α=0.9	127.6±0.6	126–128
Merge, α=0.7	125.7±1.7	122–128
Merge, α=0.55	113.1±3.4	103–121
Merge, α=0.5	85.8±4.2	76–92
Merge, α=0.45	68.0±6.3	55–81
Unrelated random factors	62.6±5.9	49–73

Mean, standard deviation, and range of the agreement count out of 128 over 36 layer-key observations; notation as in Table 5.

The DistilBERT layers all have the same square shape and rank. To check whether that setting determined the observed ordering, Table A3 repeats the transformations on the GPT-2-shaped layers. Quantization down to 3 bits, noise up to σ=0.01, removal of one of eight components, and merges retaining at least 0.55 of the original update pass in all 36 observations. Strong noise, unrelated factors, and the equal-weight merge fail in all of them. Removing four of eight components is less decisive: 23 of the 36 observations pass. The corresponding rank 4 experiment accepted removal of two components in every layer. This difference suggests that the tolerance inferred from one rank should not be transferred unchanged to adapters that distribute their update across more components.

### Appendix E.2. Tests of the Fair-Bit Model

The third test of Section 6.5 uses independently trained adapters and checks the parts of the fair-bit model separately. On the 904 Lots-of-LoRAs adapters, bit bias exists in the population. For each layer and key, between 3 and 36 of the 128 probe positions show a fraction of ones that differs significantly from 1/2 after false-discovery-rate control. Later layers have more such positions, because adapters of one base model share a common component of their updates. For fixed pairs, however, the counts over 1000 keys are consistent with Bin(m,p): the variance ratios of ten pairs lie between 0.90 and 1.11, and none is significant after correcting for the ten tests. Across all candidates of one registered adapter, the correlation between agreement indicators has mean about 0 and standard deviation 0.036, the sampling noise for 903 candidates. The cosines of a pair are strongly correlated across layers, with a mean of 0.67. The counts are only weakly correlated, with a mean of 0.03 and a maximum of 0.07, because most pairs have *p* close to 1/2. We estimate *p* for every pair and layer by its agreement rate averaged over the three keys. Inserting this measured distribution of *p* into Equation (13) gives a plug-in estimate of $4.8\times 10^{-15}$ for the adapter-level false-accept probability, against $9.3\times 10^{-15}$ from the angle law and $6.5\times 10^{-41}$ under fair bits. This value describes the measured collection under the model and does not bound the false-accept probability of adapters outside it. Even a shared p=0.70 in every layer gives only $1.7\times 10^{-4}$. Figure A2 summarizes these tests.

### Appendix E.3. Shape Counts and Proof-System Measurements

Section 6.8 asked how many circuit shapes a hub has to support and which proof system suits them. Table A4 gives the counts behind the first answer. The 40 most frequent of the 179 shapes cover two thirds of the adapter modules, while a few rare shapes, of high rank or with large MLP projections, take most of the storage. Table A5 gives the measurements behind the second answer. The universal-setup systems need no contribution per shape, but in our measurement they prove far more slowly and need larger keys.

**Table A4:** Circuit shapes for the 27 base models with the most adapters on Hugging Face, m=128. Coverage is the share of the 3501 sampled adapter modules. Proving keys are estimated at 487 bytes per constraint; each verification key has about 3.8 kB.

Shapes	Number	Coverage	Proving Keys	Verification Keys
Most frequent 10	10	27.0%	83 GB	38 kB
Most frequent 20	20	44.2%	261 GB	77 kB
Most frequent 40	40	66.2%	1.27 TB	153 kB
Most frequent 80	80	87.0%	2.99 TB	307 kB
Rank at most 64	131	94.1%	5.01 TB	503 kB
All	179	100%	20.7 TB	687 kB

**Table A5:** Three proof systems on the complete circuit at width 64, 662,302 constraints. The Groth16 setup is the initial circuit-specific key plus one contribution; the PLONK and FFLONK setups are public preprocessing on the universal powers-of-tau file. Proving and verification times are medians of three runs, and of five and 30 runs for Groth16 with rapidsnark.

System, Prover	Setup	Proof	Verify	Proof File	Proving Key	Verification Key
Groth16, rapidsnark	82 s, 3.4 GiB	1.45 s, 0.5 GiB	3.8 ms, native	706 B	0.32 GB	3.8 kB
Groth16, snarkjs	same key	11.8 s, 5.5 GiB	0.31 s	706 B	0.32 GB	3.8 kB
PLONK, snarkjs	211 s, 14.1 GiB	657 s, 16.6 GiB	0.33 s	2252 B	4.89 GB	2.0 kB
FFLONK, snarkjs	165 s, 26.7 GiB	826 s, 31.5 GiB	0.32 s	2259 B	6.50 GB	1.1 kB

### Appendix E.4. Properties of Related Systems

Table A6 complements the cost comparison of Section 6.9. It places the proof-based systems beside the fingerprinting and watermarking schemes designed for LoRA. For each system it records what the verifier sees, what stays hidden from it, and which edits or attacks its authors evaluated. Section 6.9 draws three observations from it.

**Table A6:** What the verifier learns and which transformations were evaluated. White-box: the check reads model or adapter weights; black-box: it queries the model or inspects its outputs. API: application programming interface.

System	Access	Hidden from the Verifier	LoRA- Specific	Transformations or Attacks Evaluated
ZKROWNN [25]	White-box	Trigger key and watermark; the weights are a public input	No	None beyond the underlying watermark
zkLLM [26]	API and proof	Weights (no fingerprint)	No	Not applicable
ZKLoRA [10]	White-box	LoRA weights; activations are shared	Yes	Not applicable
VeriLoRA [11]	White-box	Weights, LoRA factors, training data	Yes	Not applicable
HuRef [8]	White-box by owner	Weights; proof described, not implemented	No	Supervised and reinforcement fine-tuning, continued pretraining, weight permutation and rotation
PVMark [9]	Output text	Detection key	No	Word deletion and synonym substitution
TIM [27]	Output image	Seed and embedding positions	No	JPEG compression
NoisePrints [28]	Output image	Generation seed	No	Image processing, regeneration, a targeted inversion attack, geometric edits
ZK-WAGON [40]	Output image	Weights and prompts	No	None
FTI [12]	White-box	Base and fine-tuned weights	Rank class	Policy violations only (no provenance claim)
SEAL [32]	White-box	Nothing; the passport is disclosed in a dispute	Yes	Pruning, fine-tuning, rank reduction, ambiguity
LoRAGuard [33]	Black-box	Not addressed	Yes	Merging by addition and negation, fine-tuning, pruning
AuthenLoRA [31]	Output image	Not addressed	Yes	Image distortions, merging, pruning, quantization to int4
Merge-resistant [35]	White-box	Not addressed	Yes	Merging
LoRA-FP [36]	Black-box	Not addressed	Yes	Continued fine-tuning, pruning, merging (task arithmetic, DARE, TIES)
Chain & Hash [7]	Black-box	Nothing; questions, answers, and hash are disclosed	Partly	Meta-prompts, fine-tuning, int8 quantization, paraphrasing, collusion
ZK-AdapterMark	White-box, by the prover	Key and reference responses; in the confidential mode also the weights	Yes	Quantization (to 2 bits and NF4), noise, rank reduction, pruning, merging (linear, TIES, DARE), continued fine-tuning, and the adaptive attacks of Section 6.6
